# Application of Liquid Chromatography-Ion Mobility Spectrometry-Mass Spectrometry-Based Metabolomics to Investigate the Basal Chemical Profile of Olive Cultivars Differing in *Verticillium dahliae* Resistance

**DOI:** 10.1021/acs.jafc.4c07155

**Published:** 2024-11-22

**Authors:** Irene Serrano-García, Ioannis C. Martakos, Lucía Olmo-García, Lorenzo León, Raúl de la Rosa, Ana M. Gómez-Caravaca, Angjelina Belaj, Alicia Serrano, Marilena E. Dasenaki, Nikolaos S. Thomaidis, Alegría Carrasco-Pancorbo

**Affiliations:** 1 Department of Analytical Chemistry, Faculty of Sciences, University of Granada, Ave. Fuentenueva s/n, Granada 18071, Spain; 2 Analytical Chemistry Laboratory, Chemistry Department, National and Kapodistrian University of Athens, Panepistimiopolis Zographou, Athens 15771, Greece; 3 Food Chemistry Laboratory, Department of Chemistry, National and Kapodistrian University of Athens, Panepistimiopolis Zographou, Athens 15771, Greece; 4 IFAPA Centro Alameda del Obispo, Av. Menéndez Pidal s/n, Córdoba 14004, Spain; 5 Instituto de Agricultura Sostenible, Consejo Superior de Investigaciones Científicas, Av. Menéndez Pidal s/n, Córdoba 14004, Spain; 6 The University Institute of Research into Olives and Olive Oils (INUO), University of Jaén, Campus Las Lagunillas s/n, Jaén 23071, Spain

**Keywords:** *Olea europaea L.*, LC-MS profiling, TIMS, olive roots, olive stems, olive leaves, pathogen resistance, Verticillium wilt of olive

## Abstract

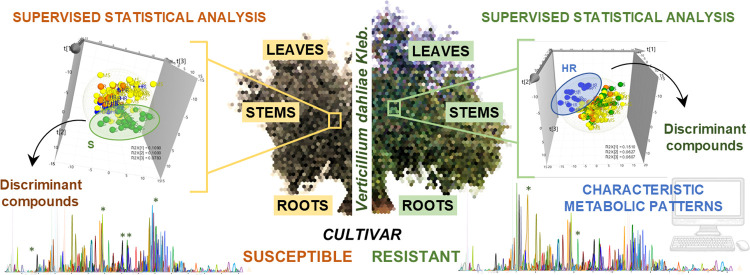

The limited effectiveness of current strategies to control Verticillium wilt of olive (VWO) prompts the need for innovative approaches. This study explores the basal metabolome of 43 olive cultivars with varying resistance levels to *Verticillium dahliae*, offering alternative insights for olive crossbreeding programmes. The use of an innovative UHPLC-ESI-TimsTOF MS/MS platform enabled the annotation of more than 70 compounds across different olive organs (root, stem, and leaf) and the creation of a preliminary compilation of ^TIMS^CCS_N2_ experimental data for more reliable metabolite annotation. Moreover, it allowed the documentation of numerous isomeric species in the studied olive organs by resolving hidden compounds. Multivariate statistical analyses revealed significant metabolome variability between highly resistant and susceptible cultivars, which was further investigated through supervised PLS-DA. Key markers indicative of VWO susceptibility were annotated and characteristic compositional patterns were established. Stem tissue exhibited the highest discriminative capability, while root and leaf tissues also showed significant predictive potential.

## 1 Introduction

*Olea europaea* L. has coexisted with mankind since prehistoric times, undergoing a lengthy process of intentional or accidental domestication.^1^ The fruit is the most valued part of the tree and, due to its profitability, its cultivation has been steadily increasing worldwide. Indeed, olive-growing area is currently 19% higher compared to the beginning of the 21st century.^2^ Concurrently, the modernization of olive management has witnessed the emergence of high-density growing systems and the widespread adoption of drip irrigation systems worldwide, with particular prominence in Andalusia, Spain.^3,4^ However, a downside of these significant changes has been the rapid spread of some pests and diseases, such as Verticillium wilt of olive (VWO), across olive-growing regions, resulting in substantial economic losses for producers.^5,6^ This severe pathology is caused by the soil-borne fungus *Verticillium dahliae* Kleb. and was first diagnosed in 1946 in Italy and, later, in the entire Mediterranean basin.^7^ Numerous factors contribute to its uncontrolled expansion, but particularly noteworthy is the fungus’s exceptional resistance, facilitated by microsclerotia, which are triggered to germinate by the root exudates of the plant.^8^*V. dahliae* penetrates the olive tree via roots and disseminates rapidly through other organs (trunk, barks, leaves, etc.) to colonize the xylem vessels causing the host plant’s water and nutrients collapse.^8^ The severity of plant symptoms will vary depending on several factors, including the type of infecting isolates, the density of inoculum, the susceptibility of the cultivar, and environmental conditions. In severe cases, this can lead to the complete death of the tree.^9,10^ Owing to the extended survival of microsclerotia and the limited efficacy of fungicides, the management of *V. dahliae* predominantly focuses on combining preventive strategies with sustainable agricultural practices.^9,11^ In this context, the use of olive cultivars possessing inherent resistance to *V. dahliae* as a component of integrated control strategies has been widely advocated to mitigate disease incidence.^9,12^ Many studies have already categorized in terms of resistance and/or susceptibility a substantial number of olive cultivars by using multiple disease parameters related to physical symptomatology and/or fungus infection rate.^13−16^ However, the key factors determining VWO resistance as a selection criterion in olive breeding programs remain unclear, and further research is required.

In plant biology, metabolomics has been pivotal in elucidating the physiological and biochemical responses of hosts to biotic and abiotic stresses.^17^ Metabolomics is categorized into targeted and nontargeted approaches, which differ mainly in the methodologies and pursued objectives. Regarding VWO, only targeted approaches have been employed so far, covering a limited section of metabolome. Thus, several secondary metabolites (mainly phenolic compounds) have been evaluated in various infected olive organs and tissues (roots, stems, cortex, xylem, etc.) to explore their role in the plant’s defense mechanisms against *V. dahliae*.^18−21^ Indeed, some of these metabolites such as rutin, oleuropein, luteolin-7-glucoside or hydroxytyrosol have been previously described to exhibit *in vitro* antifungal activity against this vascular pathogen.^19,22^ More extensively, Cardoni and coauthors determined 31 secondary metabolites belonging to simple phenols and glycosides, secoiridoids and derivatives, lignans, and triterpenic acids to evaluate major changes in metabolic profiles of infected-olive root extracts.^23^ In that work, a strong relationship between the quantitative basal metabolic profile and olive cultivar susceptibility was pointed out. Building on these findings and providing additional evidence, Serrano-García and coauthors, in a recent study, depicted the distribution of 56 basal metabolites in three olive organs, emphasizing key quantitative differences observed in relation to VWO-resistance levels.^24^ These authors also demonstrated the ability of the quantified metabolites to differentiate olive cultivars based on fungal resistance through the use of both supervised and unsupervised statistical analyses.

Although nontargeted metabolomics has not been applied in VWO-pathology to date, this holistic approach has provided valuable insights trying to elucidate the resistance mechanisms of olive tree against *Xylella fastidiosa*,^25^ cotton against *Aspergillus tubingensis*(26) or tobacco against *Phytophthora parasitica* var. *nicotianae*.^27^ The primary objective of nontargeted metabolomics is to screen the metabolome of samples exhibiting specific traits, such as resistance, as well as to identify discriminant biomarkers without prior knowledge of their identity. The most time-consuming step in this process is metabolite/marker identification, which demands careful and detailed data interpretation. Conventional LC-High Resolution MS (LC-HRMS) platforms widely used in metabolomics provide many ion descriptors (e.g., retention time, accurate mass, molecular formula, isotopic distribution, and MS/MS fragmentation). These descriptors facilitate metabolite identification by comparison with comprehensive databases and published literature. Over the past decade, the integration of ion mobility spectrometry (IMS) with HRMS has introduced an additional molecular descriptor known as the collision cross section (CCS) value. The CCS is a unique physicochemical parameter related to the size, shape, and charge of the molecules, which is measured with a specific buffer gas, pressure, and temperature.^28^ The mobility dimension enhances metabolite identification with higher confidence and improves sensitivity by reducing the signal-to-noise ratio. Additionally, it increases the selectivity of the method by boosting peak capacity.^29^ Furthermore, for isomers that cannot be resolved chromatographically or differentiated by MS, ion mobility provides an additional separation dimension, enabling the detection and potential distinction of previously hidden isomers. Due to these advantages, the use of IMS-MS in the analysis of natural products has grown significantly, becoming a valuable tool for researchers working with complex matrices.^30,31^ Consequently, IMS stands out as a powerful technique to enhance the performance of nontargeted LC-MS methods. However, there remains a notable lack of experimental CCS databases in plant metabolomics, particularly for compounds without available pure standards, such as those derived from olive matrices. Consequently, the CCS descriptor remains incompletely integrated into the workflow for metabolite characterization, and further research is needed to achieve widespread acceptance.

Being aware of the existence of a significant information gap regarding VWO disease and the capabilities of the analytical platform used, this study pursued three main objectives: (i) to evaluate the potential of the innovative UHPLC-ESI-TimsTOF MS platform to maximize metabolome information from olive-derived matrices, leading to the creation of a preliminary list of compounds based on collision cross-section values (^TIMS^CCS_N2_); (ii) characterize the presence or absence of these secondary metabolites in various olive plant organs; and (iii) apply an untargeted approach to comprehensively investigate and delineate basal metabolic differences in roots, stems, and leaves of 43 olive cultivars as a function of their resistance to *Verticillium dahliae* Kleb. infection.

## 2 Materials & Methods

### 2.1 Plant Material and Sample Pretreatment

Healthy one-year-old plants from 43 different olive cultivars obtained by vegetative propagation of semihardwood stem cuttings were provided from the World Olive Germplasm Bank (WOGBC) of Centro IFAPA “Alameda del Obispo” in Cordoba, Spain.^32^Table 1 includes the cultivars selected in the present study classified according to the VWO-resistance category.^9,14^ Plant organs (roots, stems and leaves) were sampled from three different plants of the cultivars under study, resulting thus in a comprehensive collection of 129 samples per plant organ (387 samples in total considering all the tissues). As plant pretreatment, olive organs were carefully detached from the tree, followed by thorough wet cleaning. Afterward, the detached tissues were air-dried at room temperature in a dark environment until a constant weight was achieved. The dried material was then finely powdered, homogenized to uniform particle size using a 0.5 mm metal sieve, and stored at −23 °C until further use.

**Table 1:** Olive Cultivars Included in the Study, Their Classification According to VWO-Resistance, and the Code Used for Their Identification

**category**	**olive cultivars and code used**
highly resistant (HR)	“I117–120” **(G1)**, “Frantoio” **(G2)**, “I111–2” **(G3)**, “I117–117” **(G4)**, “Manzanillera de Huércal-Overa” **(G5)**, “Empeltre” **(G6)**
resistant (R)	“Uslu” **(G7)**, “Maarri” **(G8),** “Koroneiki” **(G9)**, “Leccino” **(G10)**, “Mavreya” **(G11)**, “Dokkar” **(G12)**
medium susceptible (MS)	“Fs17” **(G13)**, “Klon 14–1812” **(G14)**, “Arbequina” **(G15)**, “UCI 2–35” **(G16)**, “Mawi” **(G17)**, “UCI 10–30” **(G18)**, “Fishomi” **(G19)**, “Changlot Real” **(G20)**, “Piñonera” **(G21)**, “UCI 2–68” **(G22)**, “Lianolia Kerkyras” **(G23)**, “Picual” **(G24)**, “Barri” **(G25)**, “Picudo” **(G26)**, “Myrtolia” **(G27)**, “Cornicabra” **(G28)**, “Barnea” **(G29)**, “Verdial de Vélez Málaga-51” **(G30)**, “Sikitita” **(G31)**, “Manzanilla de Sevilla” **(G32)**, “Morrut” **(G33)**
susceptible (S)	“Chemlal del Kabylie” **(G34)**, “Abbadi Abou Gabra” **(G35)**, “Hojiblanca” **(G36)**, “Majhol-152” **(G37)**, “Abou Salt Mohazam” **(G38)**, “Menya” **(G39)**, “Temprano” **(G40)**, “Llumeta” **(G41)**, “Jabali” **(G42)**, “Mastoidis” **(G43)**

### 2.2 Chemicals and Reagents

Double deionized water, with a resistivity of 18.2 MΩ·cm, was obtained using a Milli-Q system (Millipore, Bedford, USA). High-quality ethanol (EtOH) with a minimum purity of 99% and LC-MS grade methanol (MeOH) were supplied by Prolabo (Paris, France). ESI-L low concentration tuning mix was provided by Agilent Technologies (Santa Clara, CA, USA). The pure standards of quinic acid, hydroxytyrosol, rutin, oleuropein, maslinic acid, catechin, luteolin, luteolin-7-*O*-glucoside, apigenin, tyrosol, oleanolic acid, and verbascoside were acquired from Sigma-Aldrich (St. Louis, MO, USA), as well as the ammonium acetate salt. Mobile phases were filtered through a Nylaflo 0.45 μm nylon membrane filter (Pall Corporation (Michigan, MI, USA)) while Clarinet 0.22 μm nylon syringe filters (Bonna-Agela Technologies (Wilmington, DE, USA)) were used for extracts and pure standard mixtures. The standard solution mix used for qualitative purposes was prepared by mixing the exact amount of all pure standards mentioned above in EtOH/H_2_O (80:20, v/v) to obtain a concentration of around 15 mg/L for each compound.

### 2.3 Extract Preparation

The sample preparation followed the solid–liquid extraction protocol previously outlined by Serrano-García et al.^24^ Briefly, leaf extracts were prepared by mixing 100 mg of dried and homogenized powder with 10 mL of EtOH/H_2_O (60:40, v/v) in a 15 mL falcon tube. After 1.5 min of shaking, the falcon was introduced into an ultrasonic bath working within the range of 50–60 kHz for 30 min and centrifugated for 10 min at 9000 rpm. Once the first supernatant was removed in a dark flask, the remaining solid underwent re-extraction using 10 mL of EtOH/H_2_O (80:20, v/v) in the subsequent step, followed by 10 mL of pure EtOH in the last extraction cycle. All supernatants were combined in the same dark flask (totaling 30 mL in leaf extracts). Before injection, an additional 10-fold dilution was performed using EtOH/H_2_O (80:20, v/v). Stem and root extracts were prepared following the same protocol as described above, with the extractant agent volume reduced to 5 mL at each step, resulting in a final volume of 15 mL. A 5-fold dilution was carried out for both root and stem extracts. All extracts were stored at −23 °C until analysis.

A quality control (QC) sample was prepared for each plant organ (root, stem, and leaf) by combining aliquots from the extracts of the cultivars included in this study. These samples were utilized as instrumental controls.

### 2.4 Analytical LC-IMS-MS/MS Platform Conditions

The entire sample set was analyzed using an ultrahigh performance liquid chromatography (UHPLC) equipped with an electrospray ionization source (ESI) and coupled to trapped ion mobility spectrometry-time-of-flight system (TimsTOF Pro) powered by the latest parallel accumulation serial fragmentation (PASEF) technology from Bruker Daltonics (Bremen, Germany). According to the method proposed by Martakos et al.,^33^ analytes were eluted using an Acclaim RSLC 120 C18 column (2.1 × 100 mm, 2.2 μm) from Thermo Fischer Scientific Inc. (Waltham, MA, USA), equipped with an Acquity UPLC BEH C18 VanGuard Pre-Column (2.1 × 5 mm, 1.7 μm), and maintained at a temperature of 30 °C. The injection volume was set at 2 μL and the autosampler was kept at 4 °C throughout the sequence. Mobile phases were composed by H_2_O/MeOH (90:10, v/v) (phase A) and pure MeOH (phase B), both buffered with 5 mM ammonium acetate. The chromatographic elution conditions, including time, flow rate, and mobile phase composition, were programmed as follows: 0 min, 99.0% A and 0.2 mL/min; 1.0 min, 99.0% A and 0.2 mL/min; 3.0 min, 61.0% A and 0.2 mL/min, 14 min, 0.1% A and 0.4 mL/min, 16 min, 0.1% A and 0.48 mL/min, 16.1 min, 99.0% A and 0.48 mL/min, 19 min, 99.0% A and 0.48 mL/min, 19.1 min, 99.0% A and 0.2 mL/min; and 20 min, 99.0% A and 0.2 mL/min.

Ion mobility spectrometer operated with nitrogen (N_2_) as drift gas and 100.0 ms of ramp time, monitoring features from 0.40 to 1.37 V·s/cm^2^. The ESI operated in negative polarity and *Full Scan* mode (*m*/*z* 20–1300), with specific settings including +2500 V of capillary, −500 V of end-plate offset, 10 L/min and 220 °C of dry gas, and 2.0 bar of nebulizer pressure. Two different MS acquisition modes were employed depending on the objective pursued. Broadband collision-induced dissociation (bbCID) based on data-independent acquisition (DIA) method was employed to analyze the entire sample set, providing enhanced sensitivity. Additionally, PASEF, which relies on data-dependent acquisition, was exclusively utilized in certain QC samples to generate the auto MS/MS fragmentation pattern. In this latter mode, the same precursor ion was selected and fragmented several times to generate multiple MS^2^ spectra. The software used for system control included Compass Hystar and Otof Control, supplied by Bruker Corporation. Data Analysis 5.3 software was applied to examine the acquired chromatograms.

### 2.5 System calibration, System stability assurance, and Data processing

Before starting any sequence, both TIMS and MS systems were subjected to external calibration using sodium formate and commercial ESI-L Low Concentration Tuning Mix solutions. In addition, a freshly prepared mixture (3:1, v/v) of these solutions was constantly infused to serve as internal calibration for data processing. For successful calibration, at least three reference *m*/*z* and ion mobility values from the calibration solution had to correspond with those measured in the system. The QC sample was analyzed every 10 samples to evaluate the stability of the instrument response. Additionally, pure solvent (MeOH) injections were performed at the same intervals to clean the column and ensure it remained free of contamination.

Data processing was conducted using the MetaboScape 2023 software, which utilized the T-Rex 4D (LC-TIMS-QTOF MS) algorithm to automatically recalibrate the acquired MS data. This involved conducting molecular feature selection, filtering, and scaling. Key parameters were configured during processing, such as setting the minimum extracted features by the number of occurrences to #3 for each group (in this case, for each cultivar) to ensure consistent feature presence across all cultivar replicates. The intensity threshold for peak detection was established at 1000 counts and the minimum 4D peak size was set at 100 points, while recursive features were defined at 75 points. An EIC correlation of 0.8 related to ion deconvolution was applied. The primary ion was [M-H]^−^, with [M+Cl]^−^ as seed ion and [M–H-H_2_O]^−^ and [M+CH_3_COO]^−^ as common ions. During data processing, the Within-Batch Correction tool was utilized to address potential drifts that may have occurred during the sequences. Extracted features from solvent analyses were automatically excluded if the analysis/solvent ratio exceeded 3.0. Following this, the extracted features were characterized using a number of tools that are integrated into MetaboScape. These tools include (i) SmartFormula, which derives the molecular formula of each annotated compound based on its accurate mass and isotopic pattern, taking into account any detected adducts; (ii) Compound Crawler, which searches molecular structures for specified molecular formulas in local (AnalyteDB) and online public databases (ChEBI, ChemSpider and PubChem); and (iii) MetFrag, which performs *in silico* fragmentation of potential structures and compares them with acquired MS/MS spectra. The software also supports annotation by comparing with previously established analyte lists and MS/MS spectral libraries (such as Bruker Sumner MetaboBASE Plant Library or public MS/MS databases). Typical bioactive compounds primarily consist of carbon, hydrogen, and oxygen. Therefore, our focus was on annotating compounds containing these elements, aiming for errors below 5 ppm. Additionally, the software provides a CCS prediction tool, crucial for ensuring high-reliability analyte characterization.

### 2.6 Statistical Analysis

SIMCA v14.1 software was used to perform both unsupervised principal components (PCA) and supervised partial least-squares-discriminant analysis (PLS-DA). The data matrix included 129 samples (observations) and contained all the detected features (variables) expressed as peak intensity for each olive organ type. Standard data normalization and unit variance (UV) scaling were implemented as preprocessing methods. PCA was conducted to investigate data quality, biological diversity, and natural clustering of samples based on VWO-resistance. Hotelling’s T2 (95%) and DModX (DCrit 0.05) plots were examined to detect any potential outliers within the multidimensional space of PCA. Following a thorough examination of the LC-MS data, a supervised PLS-DA statistical analysis was employed to further explore the characteristic metabolic patterns associated with the most VWO-resistant/susceptible olive cultivars. The quality of PLS-DA models was evaluated with a cross-validation test through the *R*^2^*X*, *R*^2^*Y*, and *Q*^2^ parameters. These parameters indicate the fraction of explained variance in the *X* and *Y* matrices and the predictive capability of the model, respectively. Additionally, permutations plot with 100 iterations were carried out to assess the class discrimination performance by comparing the goodness of fit (*R*^2^ and *Q*^2^) of the original model with randomly generated models where the order of *Y*-observations was permutated while keeping *X*-matrix intact.

## 3 Results & Discussion

### 3.1 Screening of Olive Organs Profiles to Build a Preliminary ^TIMS^CCS_N2_ Database

The limited availability of experimental CCS-libraries remains an unresolved obstacle to integrating ion mobility into metabolomics studies. Therefore, the initial step of this investigation was to conduct a preliminary screening of the LC-IMS-MS metabolic profiles of olive-derived matrices, aiming to build an exploratory ^TIMS^CCS_N2_ data compilation. Over 70 metabolites were annotated in the olive tree organs, including organic acids, iridoids, coumarins, simple phenols, lignans, secoiridoids, flavonoids, pentacyclic triterpenes, and their derivatives. The annotated constituents are listed in Table 1 of Supporting Information (Table S1) including the proposed compound name, chemical family, calculated molecular formula, retention time (Rt), experimental *m*/*z*, error of the mass prediction (ppm), mSigma value, ^TIMS^CCS_N2_ value, and the main MS/MS fragments observed. All data presented in Table S1 are expressed as deprotonated form [M-H]^−^, as this was the most commonly detected ion in negative polarity. In some cases, other ions such as [M+Cl]^−^, [M–H-H_2_O]^−^, and [M+CH_3_COO]^−^ were also monitored, although they were not included in the table information to contain the size of the table. The proposed compounds were cross-checked with relevant comprehensive studies focused on the in-depth characterization of olive-derived matrices to ensure their identity or confirmed using Bruker spectral libraries.^23,24,34−36^ The ion mobility descriptor was used to support metabolite identification whenever a standard was available, or if the compounds were described in the plant metabolomics ^TIMS^CCS_N2_ library generated by Schroeder and collaborators in a previous work,^37^ or in other works applying TIMS mobility.^38,39^ Additionally, it was used to propose a candidate if the predicted CCS value was consistent with the putative annotation.

Therefore, the integration of IMS has proven to be crucial in the discrimination of numerous isomeric metabolites within the matrices under study. Notably, several of these metabolites were annotated for the first time in this study. This breakthrough may be attributed to the fact that, until now, LC-MS has primarily provided isomer differentiation based solely on retention time and accurate mass. In specific cases, hidden isomers were distinguishable within a single chromatographic peak solely through the IMS dimension. Furthermore, TIMS has shown its capability to effectively separate widely overlapping peaks that cannot be entirely resolved based only on retention time and accurate mass. This capability is especially crucial for quantitative applications and represents a significant enhancement for targeted studies. The detailed workflow utilized in both scenarios is described in the following section, along with an examination of the distribution of the annotated metabolites throughout the olive tree.

#### 3.1.1 Exhaustive Qualitative Characterization of the Annotated Compounds within the Metabolome of Olive Root, Stem, and Leaf Samples

In accordance to previous studies, the qualitative metabolic profile is closely linked to the olive organ assessed.^24,34−36^Table S1 lists the metabolites that were consistently detected in all tested cultivars of each matrix. The table, as specified in the previous section, includes relevant information for each of the substances considered. As expected, most of the compounds annotated are of phenolic nature, such as simple phenols, secoiridoids, flavonoids, etc. In the case of **organic acids**, only two metabolites of this chemical class were annotated: quinic acid (C_7_H_12_O_6_) with a CCS of 134.3 Å^2^, and citric acid (C_6_H_8_O_7_) with 126.5 Å^2^. These compounds were consistently present in all organs under investigation. Three instances of **iridoids** (compounds characterized by a six-membered ring containing an oxygen bound to a cyclopentane ring) were annotated in the ethanolic extracts. Loganic acid (375.1296 *m*/*z*), with a CCS of 184.7 Å^2^, was found in the three organs examined. It is characterized by the calculated molecular formula C_16_H_24_O_10_ and shows a fragmentation pattern with MS signals of certain intensity at 213.0764, 169.0876, 151.0752, 125.0606, 113.0244, and 107.0499 *m*/*z*. The metabolites annotated as 11-hydroxyiridodial glucoside pentaacetate (555.2082 *m*/*z*; 222.3 Å^2^) and 7-deoxyloganic acid (359.1347 *m/z;* 182.4 Å^2^) were exclusively detected in roots and stems. The latter finding is not entirely in line with the results reported by Michael and coauthors, who observed the presence of 7-deoxyloganic acid exclusively in root extracts of “Koroneiki” and “Chetoui” cultivars.^36^ Serrano-Garcia and co-workers also found 7-deoxyloganic acid only in roots in a recent paper working with 10 cultivars.^24^ These differences can be easily explained, taking into account the cultivars considered in each study and the analytical methodologies employed. Two metabolites belonging to the **coumarins group** were also found in the olive-derived tissues. Aesculin (C_15_H_16_O_9_; 174.6 Å^2^), also known as esculetin hexoside, was found exclusively in olive roots and stems. The fragmentation pattern of this compound revealed the detachment of the sugar moiety, releasing its aglycone at *m*/*z* 177.0192. In contrast, aesculetin (C_9_H_6_O_4_; 127.5 Å^2^), a dihydroxycoumarin, was detected in roots, stems and leaves, and exhibited MS fragmentation with signals at *m*/*z* 149.0244, 133.0300, 105.0345, and 89.0401. Both metabolites had been previously documented in various matrices derived from olive trees.^34,36^

In general, **simple phenols and derivatives** were distributed throughout the plant, with most of them being detected in the three organs under study, although some exceptions were observed. For example, hydroxytyrosol (153.0557 *m*/*z*; 128.8 Å^2^) was detected in leaves and stems but it was not found in roots in the dilutions of extracts analyzed. Contrary to our results, Michel and colleagues reported the presence of hydroxytyrosol in the roots of “Koroneiki” and “Chetoui” cultivars, albeit at low concentrations.^36^ Serrano-García and coauthors only quantified this simple phenol in the leaves of ten olive cultivars.^24^ Ammar et al. observed the presence of hydroxytyrosol in the wood of the olive cultivar “Chemlali”, but did not detect it in extracts of “olive leaves + stems”.^34^ In the same olive cultivar, Toumi and collaborators describe the presence of hydroxytyrosol in roots.^40^ The substance annotated as isoverbascoside (C_29_H_36_O_15_; rt 6.17 min and 223.4 Å^2^) was found only in root tissue, whereas its isomer verbascoside (rt 5.73 min and 223.2 Å^2^) was found in all organs. There were other 4 metabolites detected in the three organs: two isomers of hydroxytyrosol glycoside (C_14_H_20_O_8_), tyrosol glycoside (C_14_H_20_O_7_; 161.4 Å^2^) and phenylethyl primeveroside (C_19_H_28_O_10_: 202.2 Å^2^). The two isomers of hydroxytyrosol glucoside were annotated by observation of a dual signal peak in the mobilogram (163.1 Å^2^ and 171.8 Å^2^) accompanied by a fragmentation pattern with *m*/*z* signals at 153.055, 135.045 and 123.045; the peak at 163.1 Å^2^ proved to be the predominant one. According to the literature, one of these isomers could coincide with the hydroxytyrosol 4-*O*-glucoside previously described in olive leaves.^41^

**Figure 1:**
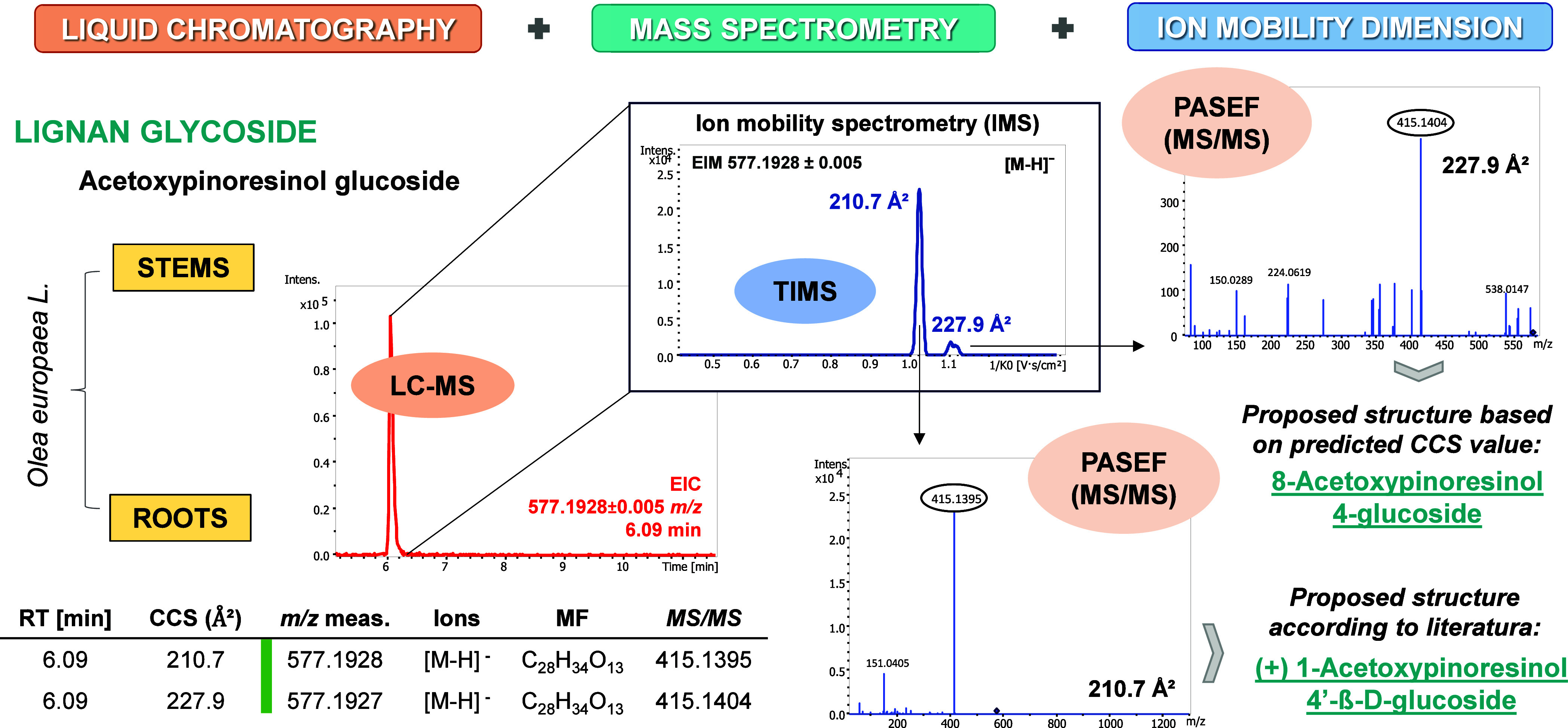
Example of the extracted ion chromatogram (EIC), mobilogram (EIM) and HRMS/MS spectra of acetoxypinoresinol glucoside to prove the potential of TIMS coupling to LC-MS/MS in the detection of hidden isomeric species without chromatographic separation.

**Lignans and derivatives** were found exclusively in olive roots and stems. However, although many reports claim the absence of this family of metabolites in olive leaves, other authors have reported the presence of trace amounts of lignans in that particular organ.^35,42^ In the present study, three potential isomers of cycloolivil glucoside (C_26_H_34_O_12_; 208.9 Å^2^, 214.5 Å^2^ and 231.4 Å^2^), two isomers of hydroxypinoresinol glucoside (C_26_H_32_O_12_; 229.5 Å^2^ and 215.9 Å^2^), and two isomers of acetoxypinoresinol glucoside (C_28_H_34_O_13_; 210.7 Å^2^ and 227.9 Å^2^) have been described. In all cases, being glycosylated compounds, the HRMS/MS spectra consistently showed a loss of 162 *m*/*z*, confirming the association with a glucose unit attached to the lignan aglycone. Several of these isomeric structures, although appearing under a single chromatographic peak, could be elucidated based on the molecular descriptors of the ions and the intensity of the peaks in the ion mobility dimension, as illustrated in Figure 1. For instance, the highest signal of acetoxypinoresinol glucoside with a CCS of 210.7 Å^2^ was denoted as (+)-1-acetoxypinoresinol-4′ß-d-glucoside in agreement with the predominant structure described in the literature.^36^ In contrast, the signal of 227.9 Å^2^ would be consistent with 8-acetoxypinoresinol-4′-glucoside, based on the predicted CCS value.

The presence of (+)-1-hydroxypinoresinol 4′-ß-d-glucoside and (+)-1-hydroxypinoresinol 1′-ß-d-glucoside has been documented for these matrices.^34,36^ However, although we have detected 2 isomers, we have not been able to attribute these identities to the observed peaks, due to lack of consensus on the abundant species; further studies are essential to clarify this. Olivil (C_20_H_24_O_7_), with a CCS of 197.8 Å^2^, was annotated from the primary fragments observed by HRMS/MS, namely the *m*/*z* 360.1227, 345.1360, 327.1252, 195.0670, and 179.0713. In the case of cycloolivil (C_20_H_24_O_7_; 205.6 Å^2^) a fragmentation pattern with two clear signals at 360.1228 and 345.1358 *m*/*z* was obtained. The lignan eluting later in the chromatographic profile was 1-acetoxypinoresinol, with a molecular formula of C_22_H_24_O_8_. Drakopoulou and co-workers, in an interesting study, highlighted the presence of two isomers of acetoxypinoresinol at 203.5 Å^2^ (1-acetoxypinoresinol) and 285.5 Å^2^ (8-acetoxypinoresinol) in extra virgin olive oil.^38^ However, in our case, only the signal linked to 1-acetoxypinoresinol was detected in the root and stem extracts, with a CCS value in line with that described by the aforementioned authors. This provides a solid basis to annotate with certainty this specific conformation.

The group with the highest number of metabolites consisted of **secoiridoids and derivatives**, which are undoubtedly one of the most representative families of compounds in olive matrices. In Table S1, 27 compounds belonging to this chemical class have been described. Practically all of them were detected in olive root, stem, and leaf. Oleuropein (C_25_H_32_O_13_; 217.5 Å^2^) and some of its derivatives were among the most relevant substances of this group, including demethyl oleuropein (C_24_H_30_O_13_; 213.5 Å^2^), two potential isomers of hydroxy oleuropein (C_25_H_32_O_14_; 218.9 Å^2^ and 227.6 Å^2^), methoxyoleuropein (C_26_H_34_O_14_; 223.2 Å^2^), oleuroside (C_25_H_32_O_13_; 217.1 Å^2^) and three isomers of oleuropein aglycone (C_19_H_22_O_8_; 186.0 Å^2^, 185.2 Å^2^ and 184.8 Å^2^). Several signals detected at 701.229 *m*/*z*, with a molecular formula of C_31_H_42_O_18_, would be consistent with isomers of the glycosidic form of oleuropein or neonuzhenide (245.5, 241.8, and 248.9 Å^2^). The first two isomers were not detected in leaves, and the latter, together with oleuroside, was absent in root tissue. Another notable subgroup of secoiridoids distributed in the three matrices considered were the compounds related to elenolic acid. Their annotations were achieved by HRMS/MS analysis, revealing two isomers of aldehydic form of decarboxymethyl elenolic acid glucoside (C_16_H_26_O_10_; 188.8 Å^2^ and 188.6 Å^2^), five isomers of elenolic acid glucoside (C_17_H_24_O_11_; 192.5, 189.9, 192.1, 190.1, and 190.4 Å^2^), elenolic acid dihexose derivative (C_25_H_38_O_18_; 231.5 Å^2^), and elenolic acid dihexose (C_23_H_34_O_15_; 234.9 Å^2^). The signals detected with *m*/*z* 389.109 at 1.29 and 1.32 min, respectively, with a molecular formula of C_16_H_22_O_11_, were tentatively annotated as oleoside/secologanoside (184.6 Å^2^ and 189.5 Å^2^) displaying a fragmentation with *m*/*z* signals of 345.116, 209.044, 183.066, 121.066, and 113.025. Finally, demethyl ligstroside (C_24_H_30_O_12_: 208.8 Å^2^), nuzhenide (C_31_H_42_O_17_; 241.1 Å^2^), lucidumoside C (C_27_H_36_O_14_; 229.1 Å^2^), and ligstroside (C_25_H_32_O_12_; 214.7 Å^2^) were also consistently annotated in all the olive matrices investigated.

**Figure 2:**
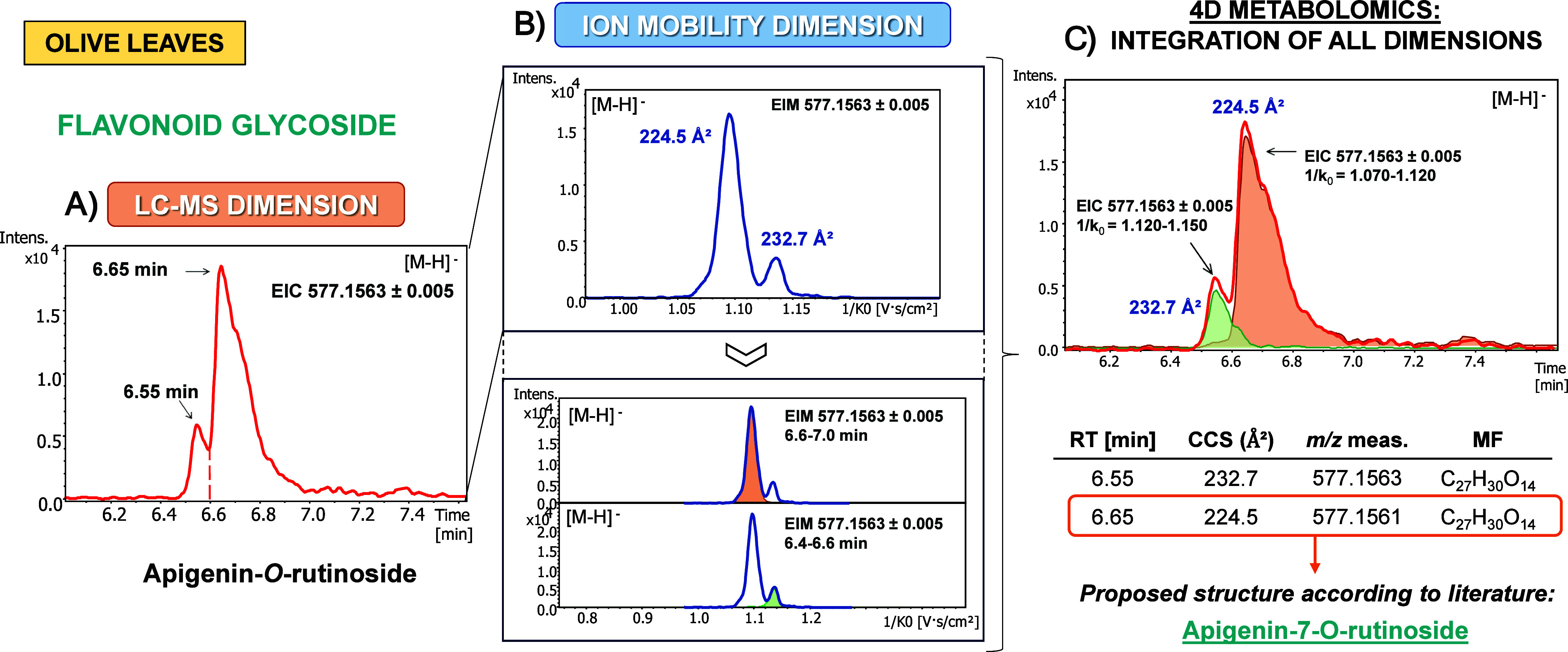
Three-step-strategy used for the complete resolution of the overlapping peaks of apigenin-*O*-rutinoside in olive leaf extracts by incorporating the ion mobility dimension into LC-HRMS/MS methodology.

**Flavonoids** proved to be another important group of phenolic compounds present mainly in olive stems and leaves. Dihydrokaempferol-*O*-glucoside (C_21_H_22_O_11_; 186.3 Å^2^), annotated through the main MS/MS fragments at 287.0550, 259.0633, 243.0664, 151.0034, and 125.0245 *m*/*z*, and two isomers of dihydroquercetin-*O*-glucoside (C_21_H_22_O_12_; 191.8 Å^2^ and 192.5 Å^2^) were found exclusively in stems organ. Their flavanonol aglycones, taxifolin (C_15_H_12_O_7_; 164.7 Å^2^) and dihydrokaemferol (C_15_H_12_O_6_; 163.4 Å^2^), were also detected exclusively in the stem extracts. Both compounds were confidently annotated as they showed a typical fragmentation pattern with MS signals at 285.0409, 177.0199, and 125.0263 *m*/*z* (for taxifolin) and 259.0598, 243.0661, 177.0561, 151.0039, and 125.0244 *m*/*z* (for dihydrokaemferol).^34−36^ Among the flavonoids that were systematically found in stem and leaf extracts, it is possible to mention the following: naringenin-*O*-glucoside (C_21_H_22_O_10_; 183.1 Å^2^), rutin (C_27_H_30_O_16_; 232.4 Å^2^), three isomers of luteolin-*O*-glucoside (C_21_H_20_O_11_; 210.2 Å^2^, 208.4 Å^2^, and 210.2 Å^2^), two isomers of quercetin-*O*-glucoside (C_21_H_20_O_12_; 202.1 Å^2^ and 210.9 Å^2^) and apigenin-7-*O*-glucoside (C_21_H_20_O_10_; 208.1 Å^2^). In all cases, the HRMS/MS spectra of these glycosylated compounds revealed a cleavage of the sugar (−162 *m*/*z*), releasing the aglycone form. It is worth noting that the following 4 compounds only appeared in the olive leaf samples: two isomers of apigenin-*O*-rutinoside (C_27_H_30_O_14_; 232.7 Å^2^ and 224.5 Å^2^), diosmin (C_28_H_32_O_15_; 231.8 Å^2^) and chrysoeriol-7-*O*-glucoside (C_22_H_22_O_11_; 215.9 Å^2^). The two isomers of apigenin-*O*-rutinoside could not be fully distinguished and annotated by LC-MS. However, relying on the TIMS dimension and following the strategy illustrated in Figure 2, both peaks were fully differentiated. Briefly, Figure 2A shows the extracted ion chromatogram (EIC) of *m*/*z* 577.1563 with a clear shoulder to the left of the main peak (min 6.55 and 6.65). Due to the absence of complete chromatographic separation for this glycosylated flavonoid, an isomeric profile scan was performed in the mobility dimension (Figure 2B). As expected, two distinct peaks emerged at 577.1563 *m*/*z* in EIM, indicating the possible presence of an isomer, as hinted above. Subsequently, specific mobility values for each segment of the coeluted peak were evidenced by locking the elution time (*m*/*z* 577.1563; min 6.4–6.6 and 6.6–7.0) in EIM. HRMS/MS spectra generated by PASEF revealed fragmentation at 269.0458 *m*/*z*, providing useful extra information for the annotation of the metabolites. Finally, by meticulous re-extraction of the features by imposing mobility constraints on the EIC, the initial overlap of the apigenin-*O*-rutinoside isomers was unraveled (Figure 2C). The predominant peak (224.5 Å^2^) was assigned as apigenin-7-*O*-rutinoside according to described in the literature.^43−45^ To conclude the overview of the described substances belonging to the flavonoid family, three metabolites were detected in all organs of all varieties: the flavanone naringenin (C_15_H_12_O_5_; 163.0 Å^2^), and two flavones, luteolin (C_15_H_10_O_6_; 160.6 Å^2^) and apigenin (C_15_H_10_O_5_; 157.6 Å^2^).

Regarding **pentacyclic triterpenes**, maslinic acid (C_30_H_48_O_4_; rt 13.13 min and 223.4 Å^2^), betulinic acid (C_30_H_48_O_3_; rt 14.00 min and 220.1 Å^2^) and oleanolic acid (C_30_H_48_O_3_; rt 14.14 min and 220.7 Å^2^) were also registered in all analyzed parts of olive tree.

### 3.2 Nontargeted Metabolomics for the Annotation of Potential Markers Related to VWO-Resistance Level on the Basal Metabolic Profiles of Olive Root, Stem, and Leaf Tissues

To investigate the potential association between VWO resistance levels and the basal metabolic profiles of olive organs, all LC-TIMS-HRMS/MS data, extracted as detailed in Section 2.5, were thoroughly analyzed using multivariate statistical approaches. Initially, unsupervised PCA was employed to assess data quality, biodiversity, and natural clustering of samples by matrix; however, this analysis did not reveal a clear natural clustering between groups. Despite this, Hotelling’s T2 (95%) and DModX (DCrit 0.05) plots were carefully evaluated to detect potential outliers across the multidimensional PCA space. Subsequent tests determined that the distant positioning of the suspected outliers was attributed to the inherent heterogeneity of biological specimens. In every instance, these samples remained in proximity to their biological replicates and their exclusion did not enhance the model. The PCA model for roots was represented by PC2 and PC3, explaining 9.28 and 7.74% of the variance, respectively. In stems, PC1 and PC9 explained 16.9 and 2.84% of the variance, while for leaves, PC2 and PC3 explained 9.26% and 7.59%. Although the natural groupings observed with the PCA models were not distinctly clear–there was no evident separation between resistance categories–, the results revealed metabolic differences between most of the evaluated cultivars (Figure S1).

**Figure 3:**
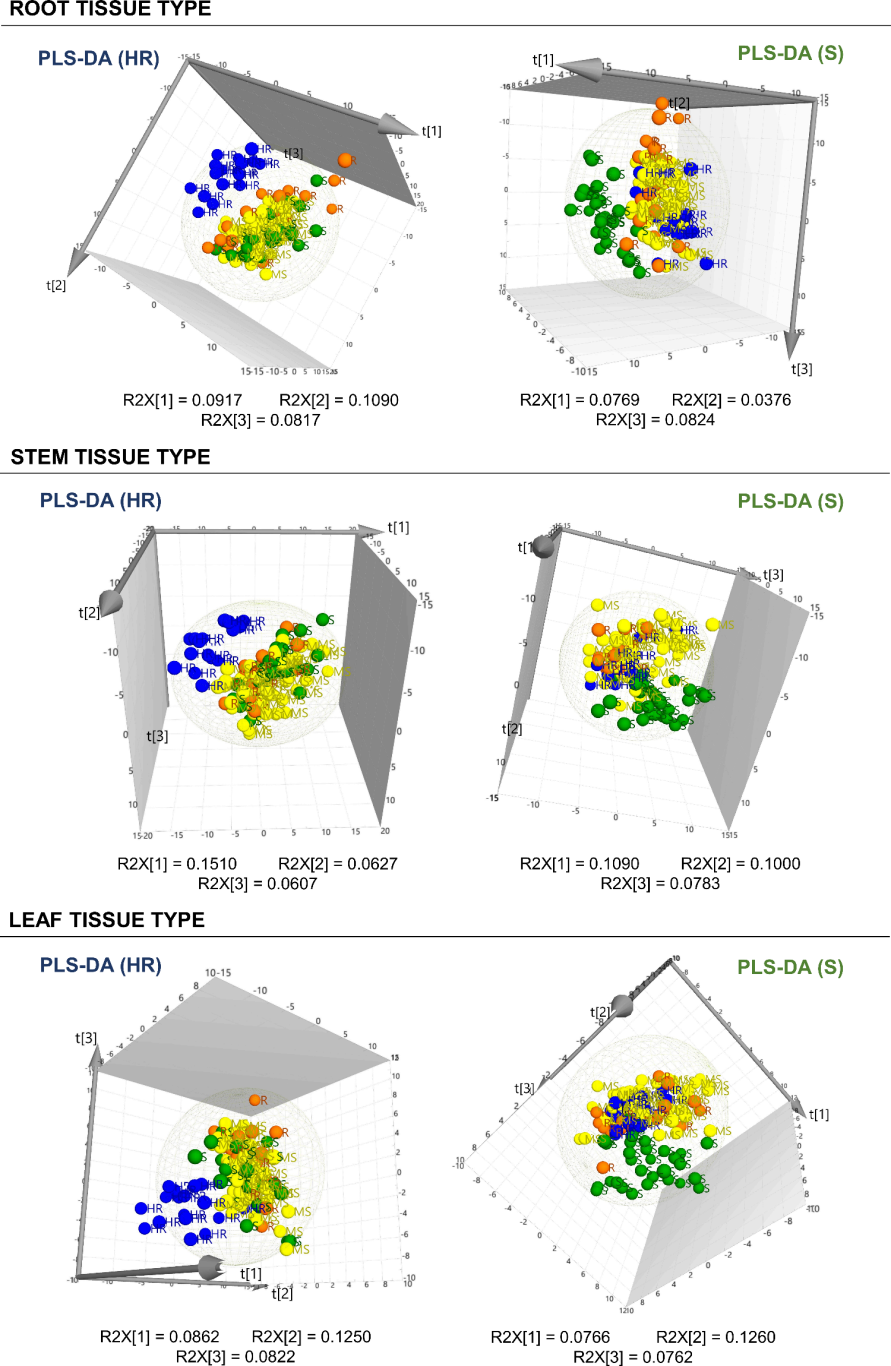
Two-class PLS-DA models and permutation tests of olive root, stem and leaf tissues for the discrimination of highly resistant (HR) and susceptible (S) cultivars to *V. dahliae*. Dots in PLS-DA plots represent different cultivars: highly resistant (HR: blue), resistant (R: orange), medium susceptible (MS: yellow), susceptible (S: green).

Therefore, the LC-IMS-MS data were second subjected to a supervised PLS-DA to determine the markers that could presumably serve to describe the characteristic metabolic patterns. By focusing on the extremes of resistance/susceptibility to VWO, two-class PLS-DA models discriminating the highest (HR) and lowest (S) resistance level versus the other cultivars were constructed for root, stem and leaf. All the PLS-DA-score plots generated in three dimensions (3D) are represented in Figure 3 using the first three principal components (3PCs). Model quality descriptors (*R*^2^*X*, *R*^2^*Y*, and *Q*^2^) are detailed in Figure S2 along with the permutation tests. Adequate linear regression parameters and predictive capacity were obtained in almost every model (*R*^2^*Y* ≥ 0.7 and *Q*^2^ ≥ 0.4).^46^ In addition, the differences of *R*^2^*Y* and *Q*^2^ values ranged between 0.2 and 0.3, which ensures that the models are not being overfitted. The PLS-DA model differentiating leaves of susceptible cultivars deviates somewhat from these reference values, although the permutation test results underline the robustness of the model in all cases, as shown by the lower positioning of *Q*^2^ (blue) and *R*^2^ (green) points on the left compared to those on the right. Moreover, the regression line of the *Q*^2^ points intersected the vertical axis at zero or below, reiterating the results of the quality tests above-mentioned. When analyzing each olive organ individually, stems led to the best cross-validation values in both models. HR cultivars vs the rest provided the best fitting model considering only 3PCs as optimal (*R*^2^*Y* = 0.803; *Q*^2^ = 0.677) while for S cultivars vs the rest, 5PCs were necessary to reach similar values (*R*^2^*Y*= 0.861; *Q*^2^= 0.630). Roots and leaves, although showing a slightly lower discriminatory efficiency, still exhibited acceptable performance.

After validating the PLS-DA models, the most influential features were selected based on their variable importance in the projection (VIP) scores. Features with the highest relevance in these models (VIP above 1.50) have been highlighted as possible class markers and have been included in Table 2, together with their associated retention time, calculated molecular formula, mass error (ppm), mSigma, ^TIMS^CCS_N2_ value, and HRMS/MS fragmentation. In addition, the regression coefficient was also included in the table to indicate the relative abundance of each compound in these categories. Positive regression coefficients indicate a peak of higher intensity, while a negative correlation indicates the opposite. The box and whisker plots of the compounds with the highest VIP values in each category are shown in Figure S3. Relevant compounds described above in olive matrices include the putative name in the table, as well as the reference to the relevant literature used for their characterization. Other substances previously described in other plant species are mentioned in the discussion when the compound could match the observed one. Some additional markers were annotated in the table through searches in HRMS/MS library and utilizing the MetFrag tool. The annotation of other important features in the models, as is typical in untargeted studies, proved to be more challenging. This underscores the need for further investigations to propose reliable candidates and facilitate annotation as accurately as possible. Those markers without identity are numbered in order of appearance in Table 2 to facilitate their recognition in the discussion. Compounds that may potentially be isomeric with others in the table are assigned with the same number for clarity.

**Table 2:** Metabolites from Olive Root, Stem, and Leaf Organs from VWO-Highly Resistant (HR) and Susceptible (S) Olive Cultivars that Exhibited the Highest Relevance in PLS-DA Models (VIP ≥ 1.50)

putative compound identity	chemical class^a^	RT (min)	m/z_exp_	molecular formula	error (ppm)	mSigma	CCS (Å^2^)	main fragments via MS/MS	R.C.	VIP	ref.
Olive Roots of HR-Cultivars											
unknown 1		12.75	277.1661	C_13_H_26_O_6_	1.62	48.4	180.3	233.1531	–0.065	2.03	
sinapyl alcohol(8- 5)coniferyl aldehyde derivate	lignans	6.95	337.1080	C_20_H_18_O_5_	–0.02	8.4	189.7	322.0871; 307.0617; 291.0652	–0.056	1.95	(45)
unknown 2 (potential isomer)		1.31	267.0722	C_9_H_16_O_9_	0.91	14.2	200.8	113.0222; 75.0088	–0.023	1.93	
vanilloyl glucoside/vanillic acid hexoside	phenolic acids	1.28	329.0876	C_14_H_18_O_9_	–0.50	9.1	183.3	167.0357; 152.0107; 123.0450; 108.0218	–0.043	1.86	(43,47)
unknown 3		4.86	313.0929	C_14_H_18_O_8_	0.15	17.4	177.6	151.0406; 150.0333	–0.051	1.82	
guaiaconic acid	lignans	6.51	339.1238	C_20_H_20_O_5_	0.13	41.4	193.8	324.1001; 310.0779; 309.0770; 281.0840	–0.034	1.75	MS/MS Lib.
methyl gallate glucoside	phenolic acids	1.35	345.0829	C_14_H_18_O_10_	0.41	43.6	172.7	-	–0.034	1.70	(48)
unknown 4		1.33	557.2084	C_22_H_38_O_16_	–0.46	13.1	223.3	389.1088; 375.1288; 213.0769	–0.043	1.67	
unknown 2 (potential isomer)		1.25	267.0721	C_9_H_16_O_9_	–0.08	15.7	151.2	113.0230; 75.0080	–0.043	1.67	
d-mannitol	sugars	1.40	181.0719	C_6_H_14_O_6_	0.67	8.9	131.1	101.0257; 85.0307; 71.0145	–0.014	1.55	(34)
unknown 5		1.39	523.1878	C_18_H_36_O_17_	–0.24	11.0	207.2	341.1091	–0.005	1.53	
lactone (ester with hydroxytyrosol)	simple phenols	6.47	321.1342	C_17_H_22_O_6_	–0.24	19.1	168.2	185.0820; 111.0823; 59.0142	0.028	1.68	(49)
unknown 6		7.37	465.2132	C_24_H_34_O_9_	0.39	28.3	208.9	-	0.051	1.63	
elenolic acid dihexose derivate	secoiridoids	4.87	625.1986	C_25_H_38_O_18_	0.05	6.9	231.5	223.0601; 179.0564; 119.0353	0.051	1.61	(23)
hydroxytyrosol glucoside derivative	simple phenols	6.81	481.2078	C_24_H_34_O_10_	–0.35	23.5	209.5	315.1078; 135.0441; 101.0231	0.049	1.56	(36)
unknown 7		1.30	237.0618	C_8_H_14_O_8_	0.66	16.3	144.8	87.0090	0.006	1.55	
unknown 8		1.31	279.0512	C_13_H_12_O_7_	0.55	24.4	151.3	207.0705; 189.0584; 115.0180	0.050	1.54	
unknown 9		6.97	569.2237	C_27_H_38_O_13_	0.13	11.0	214.6	537.1977; 403.1259; 223.0604; 121.0292	0.030	1.52	
Olive Roots of S-Cultivars											
cycloolivil glucoside (is. 3)	lignans	5.16	537.1975	C_26_H_34_O_12_	–0.27	21.0	214.5	375.1449; 195.0665; 179.0700	–0.124	2.33	(23)
d-sedoheptulose	sugars	1.27	209.0667	C_7_H_14_O_7_	0.47	10.1	139.0	85.0294; 84.0211; 78.9592; 59.1249	–0.111	1.93	(44)
unknown 10 (potential isomer)		1.30	207.0664	C_11_H_12_O_4_	–0.12	7.8	146.6		–0.061	1.63	
unknown 10 (potential isomer)		1.30	207.0661	C_11_H_12_O_4_	–0.81	13.0	178.1		–0.062	1.63	
unknown 11		6.70	199.1340	C_11_H_20_O_3_	0.09	11.3	148.1		0.078	1.64	
maslinic acid monohydroxylated derivative	pentacyclic triterpenes	11.22	487.3426	C_30_H_48_O_5_	–0.35	11.3	225.4		0.077	1.57	(35)
phenylethyl primeveroside	simple phenols	5.78	415.1609	C_19_H_28_O_10_	–0.12	4.6	202.2	149.0444	0.072	1.54	(35)
vanilloyl glucoside/vanillic acid hexoside	phenolic acids	1.28	329.0876	C_14_H_18_O_9_	–0.50	9.1	183.3	167.0357; 152.0107; 123.0450; 108.0218	0.063	1.51	(43,47)
unknown 2 (potential isomer)		1.31	267.0722	C_9_H_16_O_9_	0.91	14.2	200.8	113.0222; 75.0088	0.032	1.50	
Olive Stems of HR-Cultivars											
unknown 12		7.38	283.1187	C_14_H_20_O_6_	–0.01	13.9	166.3	199.0959; 181.0492; 139.0378; 123.0447; 99.0450; 83.0167	0.111	3.36	
elenolic acid-methyl ester	secoiridoids	5.59	255.0875	C_12_H_16_O_6_	0.28	13.3	156.4	153.0572; 101.0242; 83.0139	0.107	3.28	(35)
unknown 13		9.87	277.1804	C_17_H_26_O_3_	–0.72	26.5	171.6	233.1531; 205.1627; 59.0144	0.088	2.94	
hydroxydecarboxymethyl elenolic acid	secoiridoids	1.40	199.0609	C_9_H_12_O_5_	–0.70	20.7	138.5	155.0710	0.073	2.23	(35)
unknown 14		1.47	363.1659	C_16_H_28_O_9_	–0.46	31.5	177.2	181.0717	0.054	1.90	
unknown 15		1.33	353.0878	C_16_H_18_O_9_	0.09	19.3	185.8	191.0536; 111.0798	0.050	1.60	
dihydroquercetin-*O*-glucoside (is. 1)	flavonoids	4.67	465.1036	C_21_H_22_O_12_	–0.54	12.9	191.8	303.0505; 285.0399; 177.0191; 125.0261	–0.037	1.58	(50)
dihydrokaempferol	flavonoids	6.30	287.0560	C_15_H_12_O_6_	–0.30	2.9	163.4	259.0598; 243.0661; 177.0561; 151.0039; 125.0244	–0.020	1.51	(35)
Olive Stems of S-Cultivars											
sinapyl alcohol(8- 5)coniferyl aldehyde derivate	lignans	6.95	337.1080	C_20_H_18_O_5_	–0.02	8.4	189.7	322.0871; 307.0617; 291.0652	0.126	2.67	(45)
unknown 16		1.21	333.0623	C_16_H_14_O_8_	2.58	28.7	165.1	241.0129; 217.0518; 78.9594	0.096	1.58	
unknown 17 (potential isomer)		4.86	333.1555	C_15_H_26_O_8_	–0.78	13.1	175.9		0.084	1.51	
hydroxypinoresinol glucoside (is. 2)	lignans	6.33	535.1822	C_26_H_32_O_12_	0.25	8.9	229.5	373.1290	–0.060	1.82	(23)
hydroxypinoresinol glucoside (is. 1)			535.1820		–0.16	2.8	215.9	373.1289; 355.1189; 295.0998	–0.049	1.81	
unknown 18		7.39	315.1813	C_16_H_28_O_6_	–0.03	2.9	176.8	297.1695; 187.1334; 145.05076; 101.0605; 83.0506	–0.102	1.66	
Olive Leaves of HR-Cultivars											
unknown 19		1.39	395.1558	C_16_H_28_O_11_	–0.26	10.6	188.9	213.0771; 151.0765	0.109	2.19	
loganic acid	iridoids	1.30	375.1296	C_16_H_24_O_10_	0.07	22.7	184.7	213.0764; 169.0876; 151.0752; 125.0606; 113.0244; 107.0499	0.131	2.13	(34)
unknown 17 (potential isomer)		5.73	333.1553	C_15_H_26_O_8_	–0.45	20.4	175.1		0.123	1.65	
unknown 20 (potential isomer)		1.33	349.1502	C_15_H_26_O_9_	–0.69	38.7	217.7		0.104	1.60	
trihydroxyoctadecadienoic acid	fatty acids	7.76	327.2178	C_18_H_32_O_5_	0.13	18.7	182.3	291.1971; 229.1439; 211.1338; 171.1006	0.078	1.54	(35)
unknown 15		1.33	353.0878	C_16_H_18_O_9_	0.09	19.3	185.8	191.0536; 111.0798	0.060	1.50	
dihydroxyhexadecanoic acid	fatty acids	8.69	287.2228	C_16_H_32_O_4_	–0.06	17.0	170.3		–0.072	1.50	(35)
Olive Leaves of S-Cultivars											
1-sinapoyl-2-feruloylgentiobiose	phenolic acids	6.36	361.1041 [M–H–H]^2–^	C_33_H_40_O_18_	1.94	4.9	315.0		0.126	2.22	(51)
unknown 21		7.98	481.1361 [M–H–H]^2–^	C_44_H_52_O_24_	1.78	50.0	348.4		0.141	1.80	
naringenin	flavonoids	7.77	271.0613	C_15_H_12_O_5_	0.35	1.6	163.0	151.0028; 119.0500	0.057	1.78	(52)
dihydroxyhexadecanoic acid	fatty acids	8.69	287.2228	C_16_H_32_O_4_	–0.06	17.0	170.3		0.047	1.69	(35)
luteolin-*O*-glucoside (is. 2)	flavonoids	6.85	447.0931	C_21_H_20_O_11_	–0.45	8.3	208.4	285.0405; 133.0297	0.030	1.67	(34)
unknown 11		6.70	199.1340	C_11_H_20_O_3_	0.09	11.3	148.1		0.101	1.60	
unknown 20 (potential isomer)		1.30	349.1502	C_15_H_26_O_9_	–0.57	18.7	178.7		0.108	1.55	
coumaroyl hexoside	phenolic acids	4.95	325.0928	C_15_H_18_O_8_	–0.24	14.0	180.7	145.0292; 119.0485; 117.0340	0.084	1.54	MS/MS Lib.
apigenin 7-*O*-glucoside	flavonoids	6.73	431.0984	C_21_H_20_O_10_	–0.03	10.5	208.1	269.0459	0.032	1.53	(52)
aldehydic form of decarboxymethyl elenolic acid glucoside (is. 1)	secoiridoids	1.27	377.1452	C_16_H_26_O_10_	–0.15	8.6	188.8	197.0821; 153.0921	0.069	1.53	(45)
apigenin	flavonoids	8.80	269.0456	C_15_H_10_O_5_	0.04	6.5	157.6	225.0554; 201.0547; 149.0238	0.075	1.51	(52)
hydroxytyrosol glucoside (is. 1)	simple phenols	4.33	315.1084	C_14_H_20_O_8_	–0.31	6.4	163.1	153.0559; 135.0451; 123.0459	0.003	1.50	(45)
trihydroxyoctadecanoic acid	fatty acids	8.41	331.2488	C_18_H_36_O_5_	–0.37	17.0	180.3	157.1272	–0.010	1.65	(35)
octadecanedioic acid	fatty acids	9.86	313.2383	C_18_H_34_O_4_	–0.32	12.2	180.4	295.2291; 277.2165	–0.015	1.62	(53)
maslinic acid	pentacyclic triterpenes	13.13	471.3479	C_30_H_48_O_4_	–0.30	12.7	223.4		–0.088	1.57	(35)
trihydroxyoctadecadienoic acid	fatty acids	7.76	327.2178	C_18_H_32_O_5_	0.13	18.7	182.3	291.1971; 229.1439; 211.1338; 171.1006	–0.117	1.56	(35)

a Including glycosylated forms and derivatives within these chemical classes; R.C: regression coefficient; MS/MS Spectral Library accessed: MSMS_Public_EXP_Neg_VS17; underlined compounds indicate their previous annotation in the initial screening (Section 3.1).

Before delving into the details of Table 2, it is important to note that the distinctive compositional patterns of cultivars within a specific category depend on a combination of compounds, their relative concentrations, and potential metabolic pathways, all of which add to the complexity of the matter.

In the model developed to discriminate the **root extracts** of the **HR-cultivars** from the rest, 18 compounds were found to have significance (VIP above 1.50), with 11 of them having negative correlation coefficients (i.e., exhibiting relatively low concentrations in the HR-cultivars). Among the negatively correlated metabolites, the compound eluting at 6.95 min with a predominant MS signal with *m*/*z* of 337.1080 was tentatively annotated as a sinapyl alcohol(8- 5)coniferyl aldehyde derivate (-H_2_O, −CH_2_O), taking the previous results of Contreras et al. into account.^45^ The following compounds were also negatively correlated in the models for olive root extracts with HR cultivars: vanilloyl glucoside/vanillic acid hexoside, methyl gallate glucoside and *D-*mannitol, which were annotated on the basis of previously published reports. Also, guaiaconic acid (3,4-dimethyl-2,5-bis(4-hydroxy-3-methoxyphenyl)furan) which was annotated through the use of HRMS/MS spectral libraries. Its molecular structure and the result of MetFrag assignments to the fragments are shown in Figure S4.

Possible tentative identities could not be suggested for unknown 1 at 277.1661 *m*/*z* (C_13_H_26_O_6_). The metabolites referred to as unknown 2 (C_9_H_16_O_9_), were classified as potential isomers due to their different retention time and ^TIMS^CCS_N2_ value (200.8 and 151.2 Å). Considering that they eluted at the beginning of the chromatogram (1.31 and 1.25 min, respectively), these metabolites could be some kind of sugar derivatives, such as 3-deoxy-d-glycero-D-galacto-2-nonulosonic acid, found in roots of transgenic tobacco plants or hexosylglycerate, reported in soybean root nodules.^54,55^ To the best of our knowledge, these compounds have not been previously identified in any olive matrix and further attempts should be made to corroborate these hypotheses. The unknown 3 at 313.0929 *m*/*z* (C_14_H_18_O_8_) could be vanillin hexoside/vanilloside or *p*-hydroxyphenylacetic acid-*O*-hexoside, taking into account its fragmentation pattern. This hypothesis would be supported by the fact that both aglycones, vanillin, and *p*-hydroxyphenylacetic acid, have been found in olive extracts according to the literature,^34,35,43,56^ which could reinforce the presence of their glycosylated forms in the investigated organs. In fact, both glycosylated metabolites mentioned above have been previously described in other plant species, but so far not in olive matrices.^57,58^ Unknown 4, with MS signals at 557.2084 *m*/*z* (C_22_H_38_O_16_) showed fragments comparable to loganic acid/loganin, suggesting that it could be a derivative. Unknown 5 at 523.1878 *m*/*z* (C_18_H_36_O_17_), which was also found to be a relevant compound in this category, could not be annotated. Among the positively correlated compounds, significant metabolites annotated included a lactone (hydroxytyrosol ester), a dihexose derivative of elenolic acid and a glucoside derivative of hydroxytyrosol. In addition, a compound characterized by the predicted molecular formula C_8_H_14_O_8_ (unknown 7) could be tentatively annotated as a sugar derivative, as 3-deoxy-d-manno-octulosonate, based on its retention time and predicted CCS value. This annotation should be conclusively confirmed. The molecular formulas of three other metabolites of remarkable significance were established, with *m*/*z* values of 465.2132 (unknown 6; C_24_H_34_O_9_), 279.0512 (unknown 8; C_13_H_12_O_7_), and 569.2237 (unknown 9; C_27_H_38_O_13_). However, these substances could not be assigned specific names at this stage.

Regarding **susceptible cultivars** (**olive roots of S cultivars**), and paying attention first to the annotated compounds, a negative correlation was found for the isomer 3 of the cycloolivil glycoside (C_26_H_34_O_12_; 214.5 Å^2^). This finding is consistent with that previously observed by Serrano-García and coauthors, who also found a correlation between the root content of isomer 2 of the cycloolivil glycoside and susceptibility to *V. dahliae*.^24^ Despite the different isomer designation, we are certainly referring to the same compound, as the isomer 3 within the current work involves chromatographic separation of isomer 1, while isomer 2 coelutes with the initial peak. This may explain why the same isomers were not detected by the aforementioned authors using only LC-MS (without the mobility dimension). Another significant compound was *D*-sedoheptulose. Two possible isomeric metabolites named as unknown 10 (C_11_H_12_O_4_; 146.6 Å^2^ and 178.1 Å^2^) also showed considerable VIP values (negative correlation). In contrast, vanilloyl glucoside/vanillic acid hexoside, a maslinic acid monohydroxylated derivative, phenylethyl primeveroside and unknowns 2 (200.8 Å^2^) and 11 showed positive correlation with root extracts of susceptible olive cultivars. Unknown 11 (C_11_H_20_O_3_: 148.1 Å^2^) could be a fatty acid derivative such as 9-hydroxy-10-undecenoic acid, considering its ionization in MS, the limited *in-source* fragmentation and the predicted CCS value of this molecule. An interesting finding was that vanilloyl glucoside/vanillic acid hexoside and an isomer of unknown 2 (200.8 Å^2^) were significant at both extreme levels of resistance, underlining their paramount importance in roots for predicting, to some extent, resistance/susceptibility to VWO. According to these results, a higher presence/concentration of certain secoiridoid derivatives in olive roots together with a low content of lignan derivatives could be characteristic of highly resistant cultivars. This general statement was partially suggested by Cardoni and coauthors in a previous work.^23^

Eight compounds were pointed out as the most influential ones in the model built to discriminate between **HR-cultivars** and the rest based on **stem extracts**. Thus, HR-cultivars correlated positively with elenolic acid-methyl ester, hydroxydecarboxymethyl elenolic acid and four unidentified compounds at 283.1187 *m*/*z* (unknown 12; C_14_H_20_O_6_), 277.1804 *m*/*z* (unknown 13; C_17_H_26_O_3_), 363.1659 *m*/*z* (unknown 14; C_16_H_28_O_9_) and 353.0878 *m*/*z* (unknown 15; C_16_H_18_O_9_). In contrast, an isomer of dihydroquercetin-*O*-glucoside (is.1) and dihydrokaempferol correlated negatively in this category.

Relatively high concentrations of sinapyl alcohol(8–5)coniferyl aldehyde derivate, unknown 16 (C_16_H_14_O_8_; 165.1 Å^2^) and unknown 17 (C_15_H_26_O_8_: 4.86 min and 175.9 Å^2^), together with low quantities of hydroxypinoresinol glucoside (is. 1 and 2) and unknown 18 (C_16_H_28_O_6_; 176.8 Å^2^) characterized the stem extracts of the **susceptible cultivars** (**olive stems of S-cultivars**). Interestingly, as mentioned above, the lignan derivative sinapyl alcohol(8–5)conferyl aldehyde was also relevant for its low content in the root extracts of the HR-cultivars, highlighting the close relationship of this lignan derivative to VWO resistance/susceptibility. In summary, the distinctive compositional pattern defined for stems of highly resistant cultivars would include high levels of two elenolic acid derivatives together with low amounts of the flavonoids dihydroquercetin-*O*-glucoside (is.1) and dihydrokaempferol. In contrast, the composition of the stems of susceptible cultivars would be marked by the concentration levels of the sinapyl alcohol(8–5)conferyl aldehyde derivative and the 2 isomers of hydroxypinoresinol glucoside.

When using **olive leaf extracts,** seven compounds were found to have the highest discriminant power in the models separating **HR-cultivars** from the rest of the cultivars, while 16 possible markers were pointed out to characterize the S-cultivars. The compositional pattern of HR-cultivars was characterized by relatively high concentrations of loganic acid, trihydroxyoctadecadienoic acid, unknown 19 (C_16_H_28_O_11_; 1.39 min and 188.9 Å^2^), unknown 17 (C_15_H_26_O_8_; 5.73 min and 175.1 Å^2^), unknown 20 (C_15_H_26_O_9_; 1.33 min and 217.7 Å^2^), and unknown 15 (C_16_H_18_O_9_; 1.33 min and 185.8 Å^2^) together with low amounts of the fatty acid dihydroxyhexadecanoic acid. The unknown 20 with 349.1502 *m*/*z* had previously been documented in the metabolic profile of olive pomace, although the authors were also unable to assign an identity to it.^59^ The same occurred with unknown 17 which had been previously detected in the profiles of olive leaf but also remained as unidentified.^56^ It is worth noting that the unknown 17 detected in the leaves could be an isomer of a marker annotated in the stems of the S-varieties, since they differ only in the retention time (4.86 min in the stems and 5.73 min in the leaf extracts).

Among the 16 compounds pointed out as potential markers to define the distinctive compositional characteristics of the **S-cultivars**, 12 were positively correlated and the rest negatively correlated. Within the positive correlated biomarkers, the compound with molecular formula C_33_H_40_O_18_ was annotated as a phenolic acid derivate named 1-sinapoyl-2-feruloylgentiobiose bearing in mind the information reported by Alcántara and coauthors for olive leaf tissue as well.^51^ Coumaroyl hexoside annotated through HRMS/MS library (see Figure S4), naringenin, dihydroxyhexadecanoic acid, luteolin-*O*-glucoside (is. 2), apigenin 7*-O*-glucoside, aldehydic form of decarboxymethyl elenolic acid glucoside (is. 1), apigenin and hydroxytyrosol glucoside (is. 1) were also relevant and exhibited positive correlations; as well as a potential isomer of unknown 20, unknown 21 and unknown 11. Unknown 21 (C_44_H_52_O_24_; 7.98 min and 348.4 Å^2^) could correspond to the anthocyanidin glycoside known as peonidin-3-feruloyl sophoroside-5-glucoside; however, its identity could not be confirmed since this compound has not been previously described in extracts from olive tree tissues. The unknown compound 11 (C_11_H_20_O_3_), previously suggested to be a fatty acid, acted as a marker due to its higher contents in the leaves of susceptible cultivars. The same observation was noted in the root organ, highlighting the importance of this compound in the basal metabolic profile of olive cultivars.

In contrast, trihydroxyoctadecanoic acid, octadecanedioic acid, trihydroxyoctadecadienoic acid, and maslinic acid were negatively correlated for the S-cultivar extracts. Serrano-Garcia and colleagues reported some similarities in a previous work.^24^ They also noted the potential of hydroxytyrosol glucoside, aldehydic form of decarboxymethyl elenolic acid-glucoside and maslinic acid as markers to classify olive leaves according to VWO resistance/susceptibility. However, some discrepancies were observed about the correlation of maslinic acid and the aldehydic form of decarboxymethyl elenolic acid-glucoside in this aerial part of the olive tree. Another notable role was played by the unknown 20 (C_15_H_26_O_9_), as one of its isomers was a marker for HR-cultivars and another for S-cultivars. Moreover, dihydroxyhexadecanoic and trihydroxyoctadecadienoic acids were highlighted as markers in both resistance categories, making them compelling compounds for evaluation in the basal metabolic profiles of olive leaves. In view of the results obtained for the leaves, it can be affirmed that the precise determination of flavonoids and fatty acids would be of crucial importance to distinguish olive cultivars according to their resistance to VWO.

In conclusion, this study presents a detailed qualitative analysis of the metabolome in olive root, stem, and leaf samples utilizing a UHPLC-ESI-TimsTOF MS/MS platform. A noteworthy aspect is the preliminary but very innovative ^TIMS^CCS_N2_ database, which encompasses over 70 metabolites across various olive plant organs, thereby enhancing confidence in metabolite annotation for future research. The integration of the ion mobility dimension has also facilitated the detection and resolution of numerous isomeric species that were previously concealed within these matrices. This advancement not only enriches our understanding of the complex chemical composition of olives but also lays a solid foundation for future investigations in plant metabolomics. To further validate the CCS values established in this study, it would be necessary to continue to perform metabolomics studies that contemplate the determination of the analytes considered here in other sample sets; test more pure standards beyond those already examined; expand databases that include both experimental and predicted CCS values; and utilize a broader range of IMS instruments, including different models and brands, to ensure comprehensive validation.

Furthermore, this comprehensive study, using a nontargeted approach to roots, stems, and leaves of 43 different olive cultivars, may represent a significant achievement for the advancement of olive breeding programs. The construction of PLS-DA models allowed the identification of key markers positively and negatively correlated with HR-cultivars and S-cultivars. Stem extracts showed the highest power to categorize the resistance response. In roots, a high concentration of certain secoiridoid derivatives together with a low content of lignan derivatives could be characteristic of highly resistant cultivars. In addition, the distinctive compositional pattern defined for the stems of highly resistant cultivars would include high levels of two elenolic acid derivatives together with low amounts of the flavonoids dihydroquercetin-*O*-glucoside (is.1) and dihydrokaempferol. It can also be stated that the precise determination of flavonoids and fatty acids in leaf extracts would be of crucial importance to distinguish olive cultivars according to their resistance to VWO.

These findings offer valuable insights into the fundamental metabolic complexities linked to resistance against VWO. The integration of nontargeted metabolomics and chemometrics represents a powerful tool to unravel the chemical profiles of olive cultivars showing different levels of resistance/susceptibility to *V. dahliae* infection. Defining characteristic metabolic patterns of certain olive organs in relation to their resistance/susceptibility to VWO not only increases our understanding of the molecular mechanisms underlying basal resistance but also serves as a foundation for fine-tuning breeding strategies and enhances our knowledge on olive plant resistance.

## Supporting Information Available

^TIMS^CCS_N2_ database of many compounds detected in olive-derived matrices by LC-ESI-TimsTOF MS/MS profiling along with its presence in the olive root, stem, and leaf organ (Table S1); PCA score plots generated in two dimensions (2D) for olive roots, stems, and leaves, using the two most optimal principal components (2PCs) to effectively distinguish between HR and S samples (Figure S1); model quality descriptors (*R*^2^*X*, *R*^2^*Y*, and *Q*^2^) and permutation tests of PLS-DA models built for each tissue type (Figure S2); box and whisker plots of compounds with the highest VIP values from PLS-DA models (HR vs rest and S vs rest) for each olive organ (Figure S3); molecular structure and MetFrag outcomes for guaiaconic and coumaroyl hexoside fragment assignments (Figure S4) (PDF)
