# Supplementary material for: Application of Liquid Chromatography-Ion Mobility Spectrometry-Mass Spectrometry-Based Metabolomics to Investigate the Basal Chemical Profile of Olive Cultivars Differing in Verticillium dahliae Resistance
Source: J Agric Food Chem. 2024 Nov 22;72(49):27561–74. doi: 10.1021/acs.jafc.4c07155 (PMC11638956; doi:10.1021/acs.jafc.4c07155)
Supplement: Supplementary file 1 — jf4c07155_si_001.pdf [file jf4c07155_si_001.pdf]

SUPPORTING INFORMATION

Application of LC-ion mobility spectrometry-MS-based metabolomics to investigate the basal chemical profile of olive cultivars differing in *Verticillum dahliae* resistance

Irene Serrano-García[1], Ioannis C. Martakos[2,3], Lucía Olmo-García[1*], Lorenzo León[4], Raúl de la Rosa[4,5], Ana M. Gómez-Caravaca[1], Angjelina Belaj[4], Alicia Serrano[6], Marilena E. Dasenaki[3], Nikolaos S. Thomaidis[2], Alegría Carrasco-Pancorbo[1]

[1]Department of Analytical Chemistry, Faculty of Sciences, University of Granada, Ave. Fuentenueva s/n, 18071 Granada, Spain

[2]Analytical Chemistry Laboratory, Chemistry Department, National and Kapodistrian University of Athens, Panepistimiopolis Zographou, 15771 Athens, Greece

[3]Food Chemistry Laboratory, Department of Chemistry, National and Kapodistrian University of Athens, Panepistimiopolis Zographou, 15771 Athens, Greece

[4]IFAPA Centro Alameda del Obispo, Ave. Menéndez Pidal s/n, 14004 Córdoba, Spain

[5]Instituto de Agricultura Sostenible, Consejo Superior de Investigaciones Científicas, Av. Menéndez Pidal s/n, 14004 Córdoba, Spain

[6]The University Institute of Research into Olives and Olive Oils (INUO). University of Jaén, Campus Las Lagunillas s/n, 23071 Jaén, Spain

*Corresponding author: Dr. L. Olmo-García
Department of Analytical Chemistry
Faculty of Sciences
University of Granada
Granada, Spain.
Tel.: +34 958 249510
E-mail address: luciaolmo@ugr.es

Table S1. $^{TIMS}CCS_{N2}$ Database of many compounds detected in olive-derived matrices by LC-ESI-TimsTOF MS/MS profiling along with its presence in the olive root, stem and leaf organ.

			For experimental $[M-H]^-$					Detected in:		
**Proposed compound**	**Molecular Formula**	**Rt (min)**	***m/z* exp**	**Error (ppm)**	**mSigma**	**$^{TIMS}CCS_{N2}$ (Å^2)♦**	**Main fragments via MS/MS**	**Root**	**Stem**	**Leaf**
**Organic acids**										
*quinic acid**	$C_7H_{12}O_6$	1.24	191.0562	0.52	4.4	134.3	127.0416; 93.0344; 85.0294	x	x	x
citric acid	$C_6H_8O_7$	1.26	191.0197	-0.01	11.9	126.5	111.0092; 87.0087; 85.0291	x	x	x
**Iridoids**										
loganic acid	$C_{16}H_{24}O_{10}$	1.30	375.1296	0.07	22.7	184.7	213.0764; 169.0876; 151.0752; 125.0606; 113.0244; 107.0499	x	x	x
7-deoxyloganic acid	$C_{16}H_{24}O_9$	4.82	359.1347	-0.36	6.1	182.4	197.0814; 153.0923; 135.0817	x	x	-
11-hydroxyiridodial glucoside pentaacetate	$C_{26}H_{36}O_{13}$	5.66	555.2082	-0.06	9.9	222.3	225.1134; 183.0659; 167.0717; 151.0406	x	x	-
**Coumarins**										
aesculin	$C_{15}H_{16}O_9$	4.44	339.0721	-0.06	27.0	174.6	177.0192; 133.0299	x	x	-
aesculetin	$C_9H_6O_4$	6.39	177.0194	0.39	10.6	127.5	149.0244; 133.0300; 105.0345; 89.0401	x	x	x
**Simple phenols and derivatives**										
hydroxytyrosol glucoside (is. 1, main peak)	$C_{14}H_{20}O_8$	4.33	315.1084	-0.31	6.4	163.1	153.0559; 135.0451; 123.0459	x	x	x
hydroxytyrosol glucoside (is.2)			315.1085	0.04	2.8	171.8	153.0556; 135.0450; 123.0453	x	x	x
*hydroxytyrosol**	$C_8H_{10}O_3$	4.49	153.0557	-0.38	5.6	128.8	123.0455; 109.0293	-	x	x
tyrosol glucoside	$C_{14}H_{20}O_7$	4.69	299.1136	-0.17	10.0	161.4	137.0622; 119.0425	x	x	x
*tyrosol**	$C_8H_{10}O_2$	5.00	137.0611	1.86	3.5	127.6	119.0449; 107.0508	-	-	-
*verbascoside**	$C_{29}H_{36}O_{15}$	5.73	623.1979	-0.25	8.2	223.2	461.1663; 179.0351; 161.0242; 135.0446	x	x	x
phenylethyl primeveroside	$C_{19}H_{28}O_{10}$	5.78	415.1609	-0.12	4.6	202.2	149.0444	x	x	x
isoverbascoside	$C_{29}H_{36}O_{15}$	6.17	623.1981	0.08	5.5	223.4	461.1665; 161.0245	x	-	-
**Lignans and derivatives**										
cycloolivil glucoside (is.1, main peak)	$C_{26}H_{34}O_{12}$	5.03	537.1977	-0.09	3.8	231.4	375.1450; 195.0661; 179.0710	x	x	-
cycloolivil glucoside (is.2, main peak)			537.1976	-0.32	2.1	208.9	375.1446; 345.1372; 195.0661; 179.0716	x	x	-
cycloolivil glucoside (is.3)	$C_{26}H_{34}O_{12}$	5.16	537.1975	-0.27	21.0	214.5	375.1449; 195.0665; 179.0700	x	x	-
olivil	$C_{20}H_{24}O_7$	5.54	375.1449	-0.17	11.0	197.8	360.1227; 345.1360; 327.1252; 195.0670; 179.0713	x	x	-
cycloolivil			375.1448	-0.33	10.2	205.6	360.1228; 345.1358	x	x	-
(+)-1-acetoxypinoresinol 4'-ß-D-glucoside (is.1, main peak)	$C_{28}H_{34}O_{13}$	6.09	577.1928	0.23	8.4	210.7	415.1395	x	x	-
8-acetoxypinoresinol 4-glucoside (is.2)			577.1927	0.02	3.8	227.9	415.1404	x	x	-
hydroxypinoresinol glucoside (is.1)	$C_{26}H_{32}O_{12}$	6.33	535.1820	-0.16	2.8	215.9	373.1289; 355.1189; 295.0998	x	x	-
hydroxypinoresinol glucoside (is.2, main peak)			535.1822	0.25	8.9	229.5	373.1290	x	x	-
1-acetoxypinoresinol	$C_{22}H_{24}O_8$	7.15	415.1398	-0.02	12.0	203.5	325.1066; 280.0728; 181.0508; 151.0399; 136.0163	x	x	-
**Secoiridoids and derivatives**										
aldehydic form of decarboxymethyl elenolic acid glucoside (is.1)	$C_{16}H_{26}O_{10}$	1.27	377.1452	-0.15	8.6	188.8	197.0821; 153.0921	x	x	x
oleoside / secologanoside	$C_{16}H_{22}O_{11}$	1.29	389.1090	0.08	8.9	184.6	345.1164; 209.0446; 183.0663; 165.0519; 121.0663; 113.0250	x	x	x
oleoside / secologanoside	$C_{16}H_{22}O_{11}$	1.32	389.1088	-0.44	11.7	189.5	345.1161; 227.0558; 209.0445; 183.0669; 121.0665; 113.0253	x	x	x
elenolic acid glucoside (is.1)	$C_{17}H_{24}O_{11}$	2.14	403.1245	-0.36	15.7	192.5	371.0975; 223.0598; 181.0504; 179.0557; 127.0393; 101.0243	x	x	x
elenolic acid glucoside (is.2)	$C_{17}H_{24}O_{11}$	2.69	403.1246	-0.02	10.4	189.9	371.0978; 223.0607; 179.0561; 127.0419; 101.0238	x	x	x
aldehydic form of decarboxymethyl elenolic acid glucoside (is.2)	$C_{16}H_{26}O_{10}$	4.23	377.1452	0.02	8.8	188.6	197.0823; 153.0915	x	x	x
elenolic acid glucoside (is.3)	$C_{17}H_{24}O_{11}$	4.49	403.1245	-0.01	13.4	192.1	371.0975; 223.0601; 181.0493; 165.0555; 121.0296; 101.0241	x	x	x
elenolic acid dihexose derivate	$C_{25}H_{38}O_{18}$	4.85	625.1986	0.05	6.9	231.5	223.0601; 179.0564; 119.0353	x	x	x
demethyl oleuropein	$C_{24}H_{30}O_{13}$	4.95	525.1606	0.01	8.9	213.5	481.1694; 389.1085; 209.0442; 195.0663; 121.0670	x	x	x
elenolic acid dihexose	$C_{23}H_{34}O_{15}$	5.16	565.1775	-0.20	5.9	234.9	403.1239; 371.0981; 223.0603; 113.0224; 101.0232; 89.0232	x	x	x
demethyl ligstroside	$C_{24}H_{30}O_{12}$	5.23	509.1663	-0.22	27.1	208.8	347.1128; 277.0713; 233.0801; 165.0554; 121.0680	x	x	x
hydroxy oleuropein (is.1, main peak)	$C_{25}H_{32}O_{14}$	5.60	555.1719	0.03	15.3	218.9	537.1597; 403.1242; 393.1190; 323.0770; 151.0386	x	x	x
hydroxy oleuropein (is.2)			555.1721	-0.03	13.2	227.6	537.1595; 403.1241; 151.0383	x	x	x
neonuzhenide / oleuropein glucoside (is.1)	$C_{31}H_{42}O_{18}$	5.63	701.2293	-0.72	19.1	245.5	539.1770; 377.1241; 307.0795; 275.0925	x	x	-
elenolic acid glucoside (is.4)	$C_{17}H_{24}O_{11}$	5.94	403.1246	0.00	8.6	190.1	223.0603; 121.0289; 113.0228; 101.0246	x	x	x

**Secoiridoids and derivatives**										
nuzhenide	$C_{31}H_{42}O_{17}$	6.03	685.2347	-0.34	19.0	241.1	523.1813; 453.1391; 421.1499; 299.1132	x	x	x
neonuzhenide / oleuropein glucoside (is.2)	$C_{31}H_{42}O_{18}$	6.10	701.2297	-0.22	16.1	241.8	539.1773; 377.1238; 307.0830; 275.0925	x	x	-
methoxyoleuropein	$C_{26}H_{34}O_{14}$	6.52	569.1867	-0.55	28.2	223.2	537.1587; 403.1235; 223.0604; 165.0570; 121.0292	x	x	x
neonuzhenide / oleuropein glucoside (is.3)	$C_{31}H_{42}O_{18}$	6.54	701.2296	-0.33	19.2	248.9	539.1769; 377.1236; 307.0816; 275.0928	-	x	x
*oleuropein**	$C_{25}H_{32}O_{13}$	6.66	539.1718	0.19	4.5	217.5	403.1233; 377.1234; 307.0822; 275.0924; 223.0609; 179.0558	x	x	x
elenolic acid glucoside (is.5)	$C_{17}H_{24}O_{11}$	6.86	403.1246	-0.04	6.9	190.4	371.0967; 223.0604; 181.0483; 127.0409; 101.0249; 89.0193	x	x	x
lucidumoside C	$C_{27}H_{36}O_{14}$	7.13	583.2032	0.03	14.8	229.1	403.1224; 223.0593; 179.0541; 151.0399	x	x	x
ligstroside	$C_{25}H_{32}O_{12}$	7.26	523.1824	0.37	9.0	214.7	361.1290; 291.0879; 259.0976; 223.0621; 101.0245	x	x	x
oleuroside	$C_{25}H_{32}O_{13}$	7.35	539.1766	-0.70	10.9	217.1	403.1230; 377.1244; 345.0990; 307.0824; 275.0926; 223.0610	-	x	x
oleuropein aglycone (is.1)	$C_{19}H_{22}O_{8}$	8.03	377.1239	-0.73	3.2	186.0	307.0820; 275.0572; 149.0241; 139.0389; 111.0082; 95.0500	x	x	x
oleuropein aglycone (is.2)	$C_{19}H_{22}O_{8}$	8.33	377.1243	0.41	2.3	185.2	307.0822; 275.0564; 149.0234; 139.0390; 127.0408; 111.0088	x	x	x
oleuropein aglycone (is.3)	$C_{19}H_{22}O_{8}$	9.00	377.1242	-0.02	17.9	184.8	275.0564; 149.0237; 139.0399; 127.0407; 121.0315	x	x	x
**Flavonoids**										
dihydroquercetin-O-glucoside (is.1)	$C_{21}H_{22}O_{12}$	4.67	465.1036	-0.54	12.9	191.8	303.0505; 285.0399; 177.0191; 125.0261	-	x	-
*catechin**	$C_{15}H_{14}O_{6}$	4.80	289.0721	1.23	3.3	156.8	245.0810; 203.0744; 179.0362	-	-	-
*catechin**			289.0722	1.51	1.2	169.5	245.0811; 203.0746; 179.0358	-	-	-
dihydrokaempferol-O-glucoside	$C_{21}H_{22}O_{11}$	4.91	449.1089	0.37	10.6	186.3	287.0550; 259.0633; 243.0664; 151.0034; 125.0245	-	x	-
dihydroquercetin-O-glucoside (is.2)	$C_{21}H_{22}O_{12}$	5.40	465.1036	0.42	15.7	192.5	303.0504; 285.0406	-	x	-
taxifolin (dihydroquercetin)	$C_{15}H_{12}O_{7}$	5.77	303.0510	-0.07	3.8	164.7	285.0409; 177.0199; 125.0263	-	x	-
naringenin-O-glucoside	$C_{21}H_{22}O_{10}$	6.26	433.1138	-0.02	11.2	183.1	271.0615; 151.0018; 119.0515	-	x	x
dihydrokaempferol	$C_{15}H_{12}O_{6}$	6.30	287.0560	-0.30	2.9	163.4	259.0598; 243.0661; 177.0561; 151.0039; 125.0244	-	x	-
*rutin**	$C_{27}H_{30}O_{16}$	6.36	609.1471	1.63	2.9	232.4	301.0349; 300.0252	-	x	x
*luteolin-O-glucoside (is.1) (luteolin-7-O-glucoside)**	$C_{21}H_{20}O_{11}$	6.46	447.0934	-0.06	19.6	210.2	285.0408	-	x	x
quercetin-O-glucoside (is.1)	$C_{21}H_{20}O_{12}$	6.50	463.0879	-0.48	17.1	202.1	301.0347; 300.0251	-	x	x
apigenin-O-rutinoside (is.1)	$C_{27}H_{30}O_{14}$	6.55	577.1563	0.03	10.4	232.7	269.0458	-	-	x
apigenin 7-O-rutinoside (is.2)	$C_{27}H_{30}O_{14}$	6.65	577.1561	0.25	12.0	224.5	269.0458	-	-	x
apigenin-7-O-glucoside	$C_{21}H_{20}O_{10}$	6.73	431.0984	-0.03	10.5	208.1	269.0459	-	x	x
diosmin	$C_{28}H_{32}O_{15}$	6.74	607.1664	-0.75	24.8	231.8	299.0562; 284.0319	-	-	x
chrysoeriol-7-O-glucoside	$C_{22}H_{22}O_{11}$	6.80	461.1089	-0.25	3.7	215.9	446.0858; 299.0561	-	-	x
luteolin-O-glucoside (is.2)	$C_{21}H_{20}O_{11}$	6.85	447.0931	-0.45	8.3	208.4	285.0405; 133.0297	-	x	x
quercetin-O-glucoside (is.2)	$C_{21}H_{20}O_{12}$	6.90	463.0879	-0.71	14.7	210.9	301.0345	-	x	x
luteolin-O-glucoside (is.3)	$C_{21}H_{20}O_{11}$	7.17	447.0933	-0.02	15.4	210.2	285.0405; 284.0331	-	x	x
naringenin	$C_{15}H_{12}O_{5}$	7.77	271.0613	0.35	1.6	163.0	151.0028; 119.0500	x	x	x
*luteolin**	$C_{15}H_{10}O_{6}$	8.71	285.0407	0.89	19.3	160.6	217.0516; 199.0409; 175.0401; 151.0038; 133.0291; 107.0139	x	x	x
*apigenin**	$C_{15}H_{10}O_{5}$	8.80	269.0456	0.04	6.5	157.6	225.0554; 201.0547; 149.0238	x	x	x
**Pentacyclic triterpenes**										
*maslinic acid**	$C_{30}H_{48}O_{4}$	13.13	471.3479	-0.30	12.7	223.4	-	x	x	x
betulinic acid	$C_{30}H_{48}O_{3}$	14.00	455.3531	-0.02	2.6	220.1	-	x	x	x
*oleanolic acid**	$C_{30}H_{48}O_{3}$	14.14	455.3530	0.32	5.1	220.7	-	x	x	x

*Corroborated with a pure standard

♦The variability in the determination of CCS values, expressed as the coefficient of variation (%CV) across all samples in which each compound was detected, was consistently below 0.148%.

Principal Component Analysis

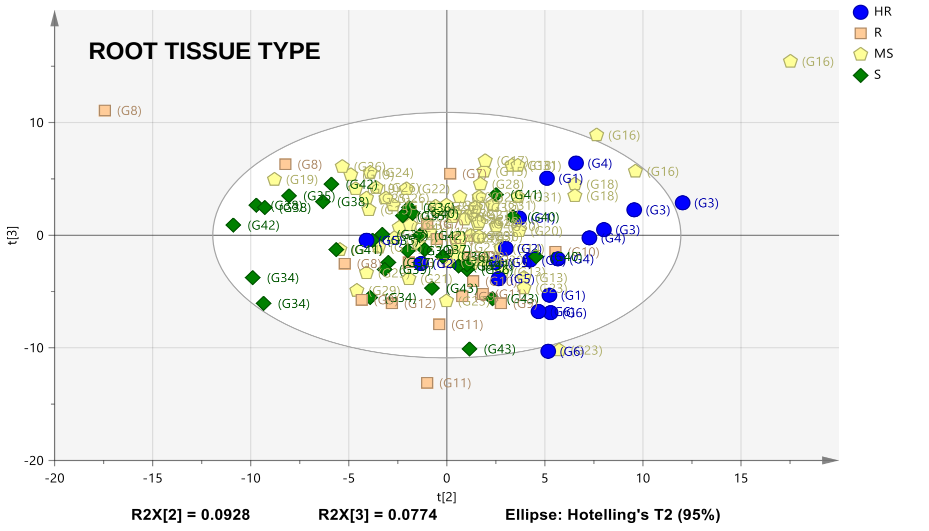

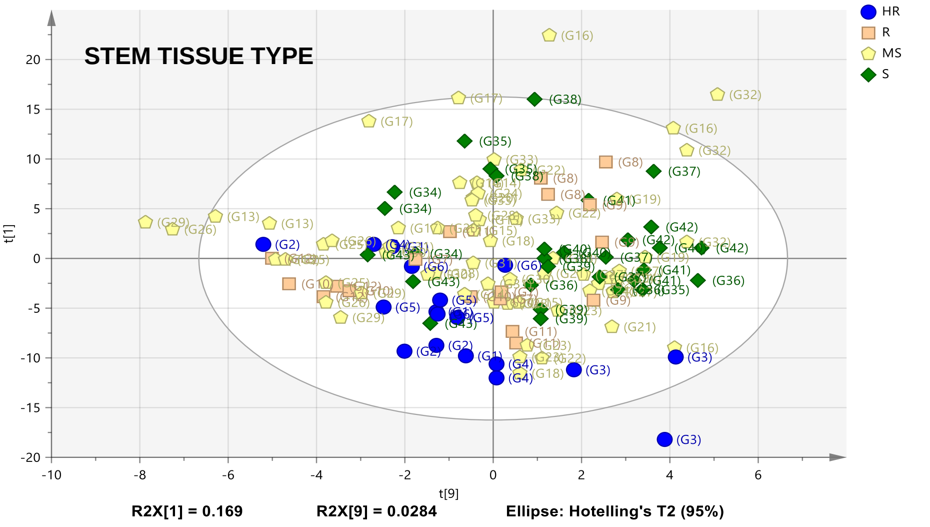

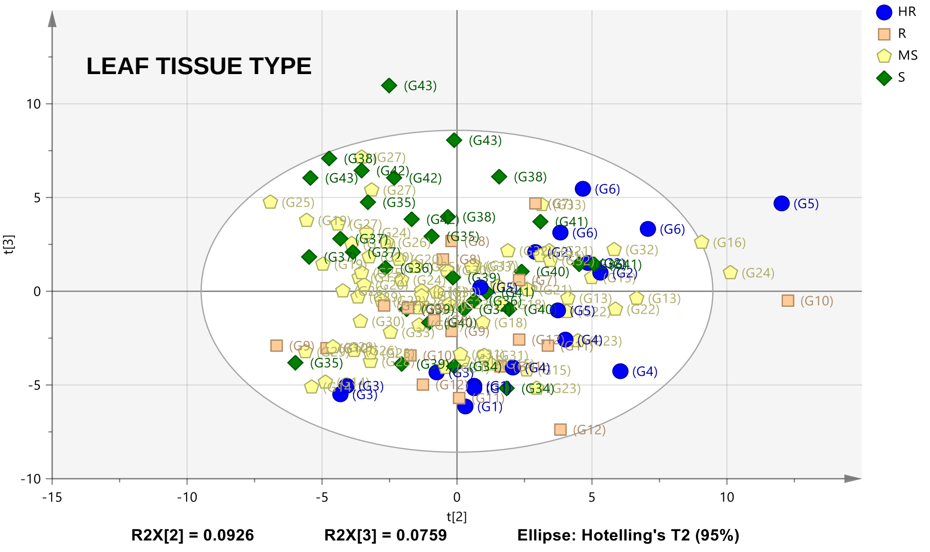

Figure S1. PCA score plots generated in two dimensions (2D) for olive roots, stems, and leaves, using the two most optimal principal components (2PCs) to effectively distinguish between HR and S samples.

ROOT TISSUE TYPE

	PLS-DA model	A	N	R^2X (cum)	R^2Y (cum)	Q^2 (cum)
(1)-	HR *vs.* Others	3	129	0.283	0.747	0.598
(2)-	S *vs.* Others	4	129	0.318	0.789	0.482

(1)- Permutations PLS-DA **(HR)**

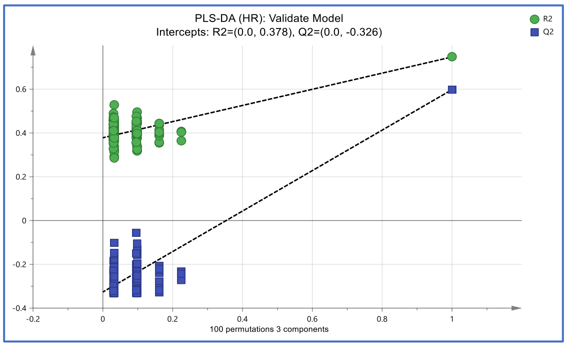

(2)- Permutations PLS-DA **(S)**

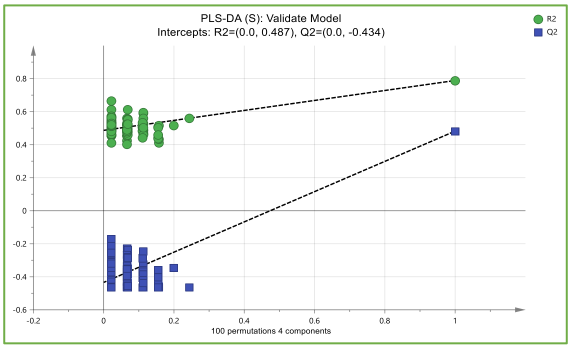

STEM TISSUE TYPE

	PLS-DA model	A	N	R^2X (cum)	R^2Y (cum)	Q^2 (cum)
(1)-	HR *vs.* Others	3	129	0.275	0.803	0.677
(2)-	S *vs.* Others	5	129	0.391	0.861	0.630

(1)- Permutations PLS-DA **(HR)**

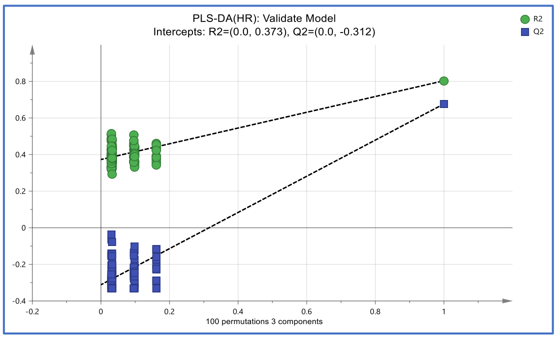

(2)- Permutations PLS-DA **(S)**

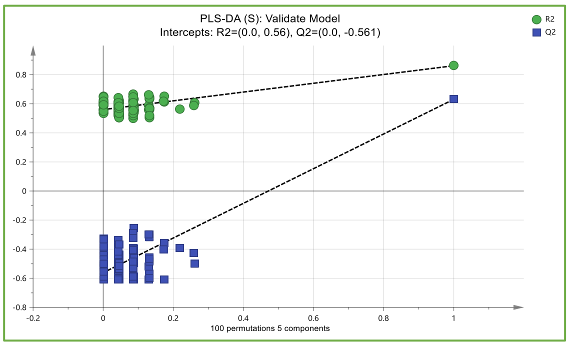

LEAF TISSUE TYPE

	PLS-DA model	A	N	R^2X (cum)	R^2Y (cum)	Q^2 (cum)
(1)-	HR *vs.* Others	4	129	0.339	0.788	0.533
(2)-	S *vs.* Others	3	129	0.279	0.666	0.318

(1)- Permutations PLS-DA **(HR)**

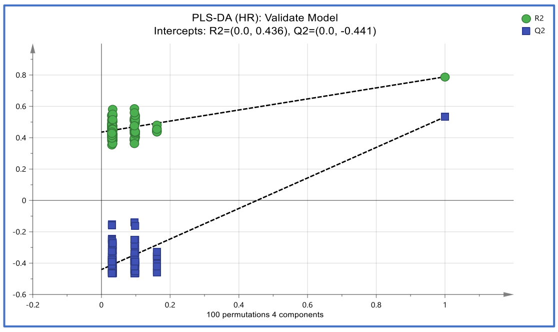

(2)- Permutations PLS-DA **(S)**

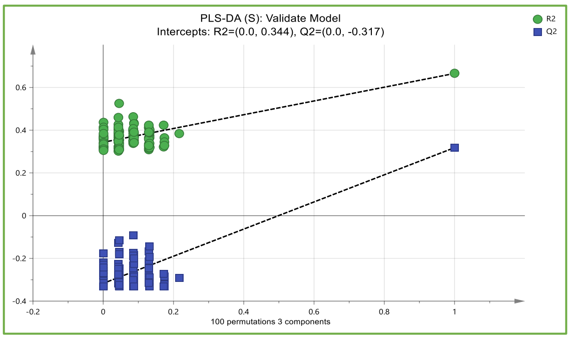

Figure S2. Model quality descriptors (R^2X, R^2Y and Q^2) and permutation tests of PLS-DA models built for each tissue type.

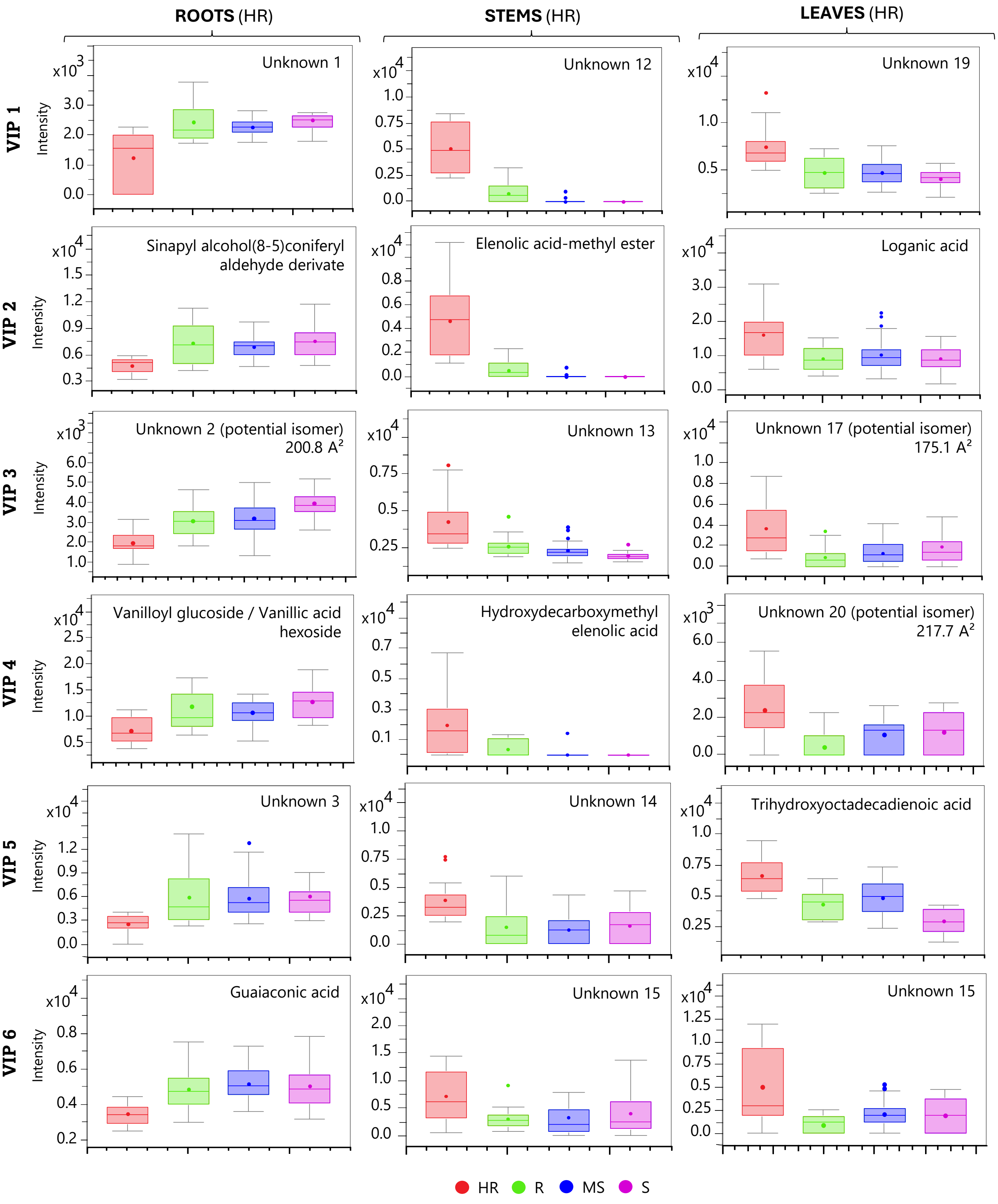

Figure S3. Box and whisker plots of compounds with the highest VIP values from PLS-DA models (HR *vs*. rest and S *vs*. rest) for each olive organ. The representation of unknown compounds that could be potential isomers include the CCS value to avoid any possible misinterpretation.

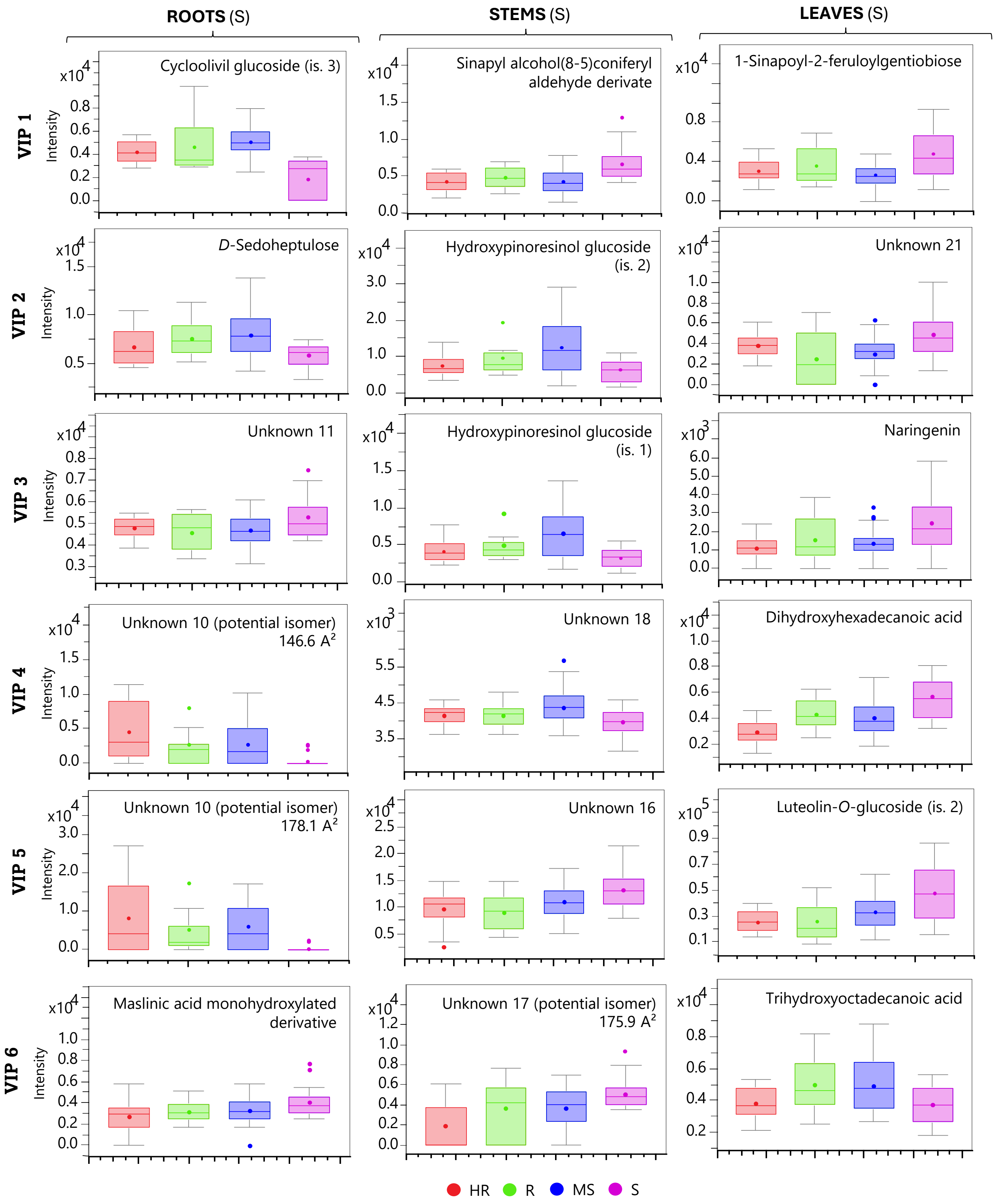

Figure S3 (cont.). Box and whisker plots of compounds with the highest VIP values from PLS-DA models (HR *vs*. rest and S *vs*. rest) for each olive organ. The representation of unknown compounds that could be potential isomers include the CCS value to avoid any possible misinterpretation.

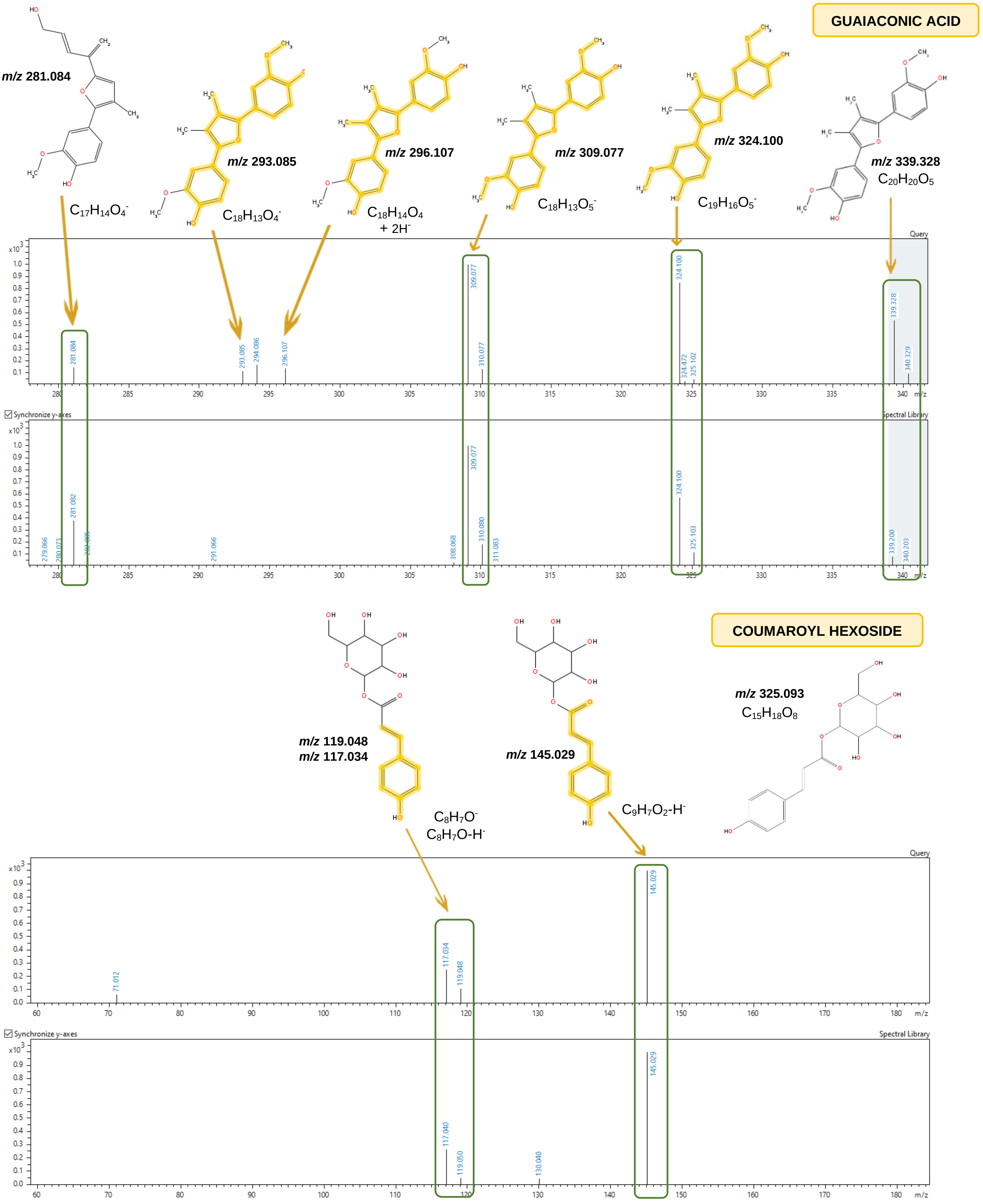

Figure S4. Molecular structure and MetFrag outcomes for guaiaconic and coumaroyl hexoside fragment assignments.
